# A Novel IgG–IgM Autoantibody Panel Enhances Detection of Early-stage Lung Adenocarcinoma from Benign Nodules

**DOI:** 10.1093/gpbjnl/qzae085

**Published:** 2024-12-11

**Authors:** Rongrong Luo, Xiying Li, Ruyun Gao, Mengwei Yang, Juan Cai, Liyuan Dai, Nin Lou, Guangyu Fan, Haohua Zhu, Shasha Wang, Zhishang Zhang, Le Tang, Jiarui Yao, Di Wu, Yuankai Shi, Xiaohong Han

**Affiliations:** Department of Clinical Laboratory, National Cancer Center/National Clinical Research Center for Cancer/Cancer Hospital, Chinese Academy of Medical Sciences & Peking Union Medical College, Beijing 100021, China; Department of Blood Transfusion, National Cancer Center/National Clinical Research Center for Cancer/Cancer Hospital, Chinese Academy of Medical Sciences & Peking Union Medical College, Beijing 100021, China; Department of Medical Oncology, National Cancer Center/National Clinical Research Center for Cancer/Cancer Hospital, Chinese Academy of Medical Sciences & Peking Union Medical College, Beijing Key Laboratory of Key Technologies for Early Clinical Trial Evaluation of Innovative Drugs for Major Diseases, Beijing 100021, China; Department of Medical Oncology, National Cancer Center/National Clinical Research Center for Cancer/Cancer Hospital, Chinese Academy of Medical Sciences & Peking Union Medical College, Beijing Key Laboratory of Key Technologies for Early Clinical Trial Evaluation of Innovative Drugs for Major Diseases, Beijing 100021, China; Department of Blood Transfusion, National Cancer Center/National Clinical Research Center for Cancer/Cancer Hospital, Chinese Academy of Medical Sciences & Peking Union Medical College, Beijing 100021, China; Department of Clinical Laboratory, National Cancer Center/National Clinical Research Center for Cancer/Cancer Hospital, Chinese Academy of Medical Sciences & Peking Union Medical College, Beijing 100021, China; Department of Clinical Laboratory, National Cancer Center/National Clinical Research Center for Cancer/Cancer Hospital, Chinese Academy of Medical Sciences & Peking Union Medical College, Beijing 100021, China; Department of Medical Oncology, National Cancer Center/National Clinical Research Center for Cancer/Cancer Hospital, Chinese Academy of Medical Sciences & Peking Union Medical College, Beijing Key Laboratory of Key Technologies for Early Clinical Trial Evaluation of Innovative Drugs for Major Diseases, Beijing 100021, China; Department of Medical Oncology, National Cancer Center/National Clinical Research Center for Cancer/Cancer Hospital, Chinese Academy of Medical Sciences & Peking Union Medical College, Beijing Key Laboratory of Key Technologies for Early Clinical Trial Evaluation of Innovative Drugs for Major Diseases, Beijing 100021, China; Department of Clinical Laboratory, National Cancer Center/National Clinical Research Center for Cancer/Cancer Hospital, Chinese Academy of Medical Sciences & Peking Union Medical College, Beijing 100021, China; Department of Medical Oncology, National Cancer Center/National Clinical Research Center for Cancer/Cancer Hospital, Chinese Academy of Medical Sciences & Peking Union Medical College, Beijing Key Laboratory of Key Technologies for Early Clinical Trial Evaluation of Innovative Drugs for Major Diseases, Beijing 100021, China; Department of Medical Oncology, National Cancer Center/National Clinical Research Center for Cancer/Cancer Hospital, Chinese Academy of Medical Sciences & Peking Union Medical College, Beijing Key Laboratory of Key Technologies for Early Clinical Trial Evaluation of Innovative Drugs for Major Diseases, Beijing 100021, China; Department of Medical Oncology, National Cancer Center/National Clinical Research Center for Cancer/Cancer Hospital, Chinese Academy of Medical Sciences & Peking Union Medical College, Beijing Key Laboratory of Key Technologies for Early Clinical Trial Evaluation of Innovative Drugs for Major Diseases, Beijing 100021, China; Department of Medical Oncology, National Cancer Center/National Clinical Research Center for Cancer/Cancer Hospital, Chinese Academy of Medical Sciences & Peking Union Medical College, Beijing Key Laboratory of Key Technologies for Early Clinical Trial Evaluation of Innovative Drugs for Major Diseases, Beijing 100021, China; Department of Medical Oncology, National Cancer Center/National Clinical Research Center for Cancer/Cancer Hospital, Chinese Academy of Medical Sciences & Peking Union Medical College, Beijing Key Laboratory of Key Technologies for Early Clinical Trial Evaluation of Innovative Drugs for Major Diseases, Beijing 100021, China; Clinical Pharmacology Research Center, Peking Union Medical College Hospital, State Key Laboratory of Complex Severe and Rare Diseases, NMPA Key Laboratory for Clinical Research & Evaluation of Drug, Beijing Key Laboratory of Key Technologies for Early Clinical Trial Evaluation of Innovative Drugs for Major Diseases, Chinese Academy of Medical Sciences & Peking Union Medical College, Beijing 100730, China

**Keywords:** Early diagnosis biomarker, Novel autoantibody, Early-stage lung adenocarcinoma, IgG autoantibody, IgM autoantibody, High-throughput protein microarray

## Abstract

Autoantibodies hold promise for diagnosing lung cancer. However, their effectiveness in early-stage detection needs improvement. In this study, we investigated novel IgG and IgM autoantibodies for detecting early-stage lung adenocarcinoma (Early-LUAD) by employing a multi-step approach, including Human Proteome Microarray (HuProt^TM^) discovery, focused microarray verification, and ELISA validation, on 1246 individuals consisting of 634 patients with Early-LUAD (stage 0–I), 280 patients with benign lung disease (BLD), and 332 normal healthy controls (NHCs). HuProt^TM^ selected 417 IgG/IgM candidates, and focused microarray further verified 55 significantly elevated IgG/IgM autoantibodies targeting 32 tumor-associated antigens in Early-LUAD compared to BLD/NHC/BLD+NHC. A novel panel of 10 autoantibodies (ELAVL4-IgM, GDA-IgM, GIMAP4-IgM, GIMAP4-IgG, MGMT-IgM, UCHL1-IgM, DCTPP1-IgM, KCMF1-IgM, UCHL1-IgG, and WWP2-IgM) demonstrated a sensitivity of 70.5% and a specificity of 77.0% or 80.0% for distinguishing Early-LUAD from BLD or NHC in ELISA validation. Positive predictive values for distinguishing Early-LUAD from BLD with nodules ≤ 8 mm, 9–20 mm, and > 20 mm significantly increased from 47.27%, 52.00%, and 62.90% [low-dose computed tomography (LDCT) alone] to 79.17%, 71.13%, and 87.88% (10-autoantibody panel combined with LDCT), respectively. The combined risk score (CRS), based on the 10-autoantibody panel, sex, and imaging maximum diameter, effectively stratified the risk for Early-LUAD. Individuals with 10 ≤ CRS ≤ 25 and CRS > 25 indicated a higher risk of Early-LUAD compared to the reference (CRS < 10), with adjusted odds ratios of 5.28 [95% confidence interval (CI): 3.18–8.76] and 9.05 (95% CI: 5.40–15.15), respectively. This novel panel of IgG and IgM autoantibodies offers a complementary approach to LDCT in distinguishing Early-LUAD from benign nodules.

## Introduction

Lung cancer remains the leading cause of cancer-related deaths worldwide, with lung adenocarcinoma (LUAD) accounting for 40% of all cases [1]. Early detection of lung cancer is crucial for reducing mortality, as patients diagnosed at stage I can achieve long-term survival rates of nearly 90% after surgery [2,3]. Low-dose computed tomography (LDCT) is the primary screening tool, but it has limitations. Approximately 50% of patients are ineligible for screening [4], while some others experience indeterminate nodules owing to the high false-positive rate of LDCT [5]. Additionally, limited resources hinder the accessibility of LDCT in low- and middle-income regions. Hence, extensive research has been dedicated to the development of biomarkers like liquid biopsies and cancer antigens. Nonetheless, achieving early lung cancer detection remains a significant challenge due to the low abundance of circulating tumor DNA in the early phase of tumorigenesis, the complexity of enrichment assays, and the instability of tumor-associated antigens (TAAs) [6,7].

Extensive evidence suggests that tumor-associated autoantibodies, immune products generated in response to oncogenic gene expression or protein aberrations, are attractive candidates for clinical applications in early cancer detection. Their early emergence in tumors, long half-life, stability in blood, and suitability for blood-based assays enhance their appeal [8]. Several commercial enzyme-linked immunosorbent assay (ELISA) kits have been developed to diagnose lung cancer by targeting combinations of autoantibodies against TAAs. In 2010, a European study first reported that a combination of autoantibodies against six TAAs, including p53, CAGE, NY-ESO-1, Annexin 1, GBU4-5, and SOX2, had a sensitivity of 36%–39% and a specificity of nearly 90% in lung cancer diagnosis [9]. The subsequent EarlyCDT^®^-Lung ELISA kit, targeting seven TAAs (p53, NY-ESO-1, CAGE, GBU4-5, SOX2, HuD/ELAVL4, and MAGEA4), demonstrated a sensitivity of 41% and a specificity of 91% [10], improving the accuracy of lung cancer nodule assessment when used alongside LDCT [11,12]. Similarly, the CancerProbe ELISA kit against seven TAAs (p53, CAGE, GBU4-5, GAGE7, SOX2, PGP9.5/UCHL1, and MAGEA1) [13] exhibited a sensitivity of 61% and a specificity of 90% in a Chinese population. Additionally, downstream analyses focused solely on evaluating the correlation between the reported autoantibody panels and various clinical characteristics of lung cancer [14,15].

Nevertheless, existing autoantibody panels necessitate the incorporation of novel autoantibodies to improve diagnostic efficacy, particularly for early-stage LUAD (Early-LUAD; stage 0–I). Although several studies have endeavored to unveil novel antibodies targeting common cancer-associated antigens or embryonic testicular antigens [16,17], the *de novo* identification of new autoantibodies remains limited. The scarcity of novel findings stems from the difficulty and complexity of conventional discovery techniques like serological proteomic analysis (SERPA), serological analysis of recombinantly expressed cDNA clones (SEREX), and phage display techniques [9,16,18]. Emerging high-throughput protein microarrays offer a promising solution, enabling the simultaneous analysis of thousands of immobilized recombinant proteins with multiple plasma samples. Nevertheless, the fabrication of microarrays encompassing a vast repertoire of recombinant proteins remains challenging. Human Proteome Microarray (HuProt^TM^), a microarray containing nearly 20,000 proteins covering approximately 85% of the human proteome, has proven efficacy in detecting new autoantibodies across various diseases [19,20].

Furthermore, different types of autoantibodies reflect distinct aspects of cancer immune surveillance [21–23]. IgG, the most predominant component of the humoral immune response [24], has been the most studied in early-stage lung cancer. IgA, crucial for mucosal immunity [25], has been investigated but appears insufficient for diagnosing lung cancer on its own [22,26–28]. However, when combined with IgG, it may enhance early detection [23]. IgM, the first antibody produced in response to TAAs [29], theoretically holds promise as a biomarker for early cancer detection. A previous study reported an accuracy of 83.02% for a five-IgM-autoantibody panel in Early-LUAD diagnosis, but larger-scale validation is needed [30]. Considering the limited research on IgM, a combined IgG–IgM approach is proposed to leverage the abundance of IgG and the early detection potential of IgM to improve the detection of early-stage lung cancer.

Therefore, this study employed a discovery-verification-validation strategy to identify novel IgG and IgM autoantibodies in 1246 individuals across three independent cohorts for Early-LUAD detection. Utilizing the HuProt^TM^ screening for discovery and a stepwise process for validation, we identified eight IgM and two IgG autoantibodies that effectively distinguished Early-LUAD from benign lung disease (BLD) with a sensitivity of 70.5% and a specificity of 77%. This novel IgG–IgM autoantibody panel significantly improved the positive predictive value (PPV) for distinguishing malignant from benign nodules, with an estimated improvement of 20%–30%.

## Results

### Study design and cohorts

**Figure 1:**
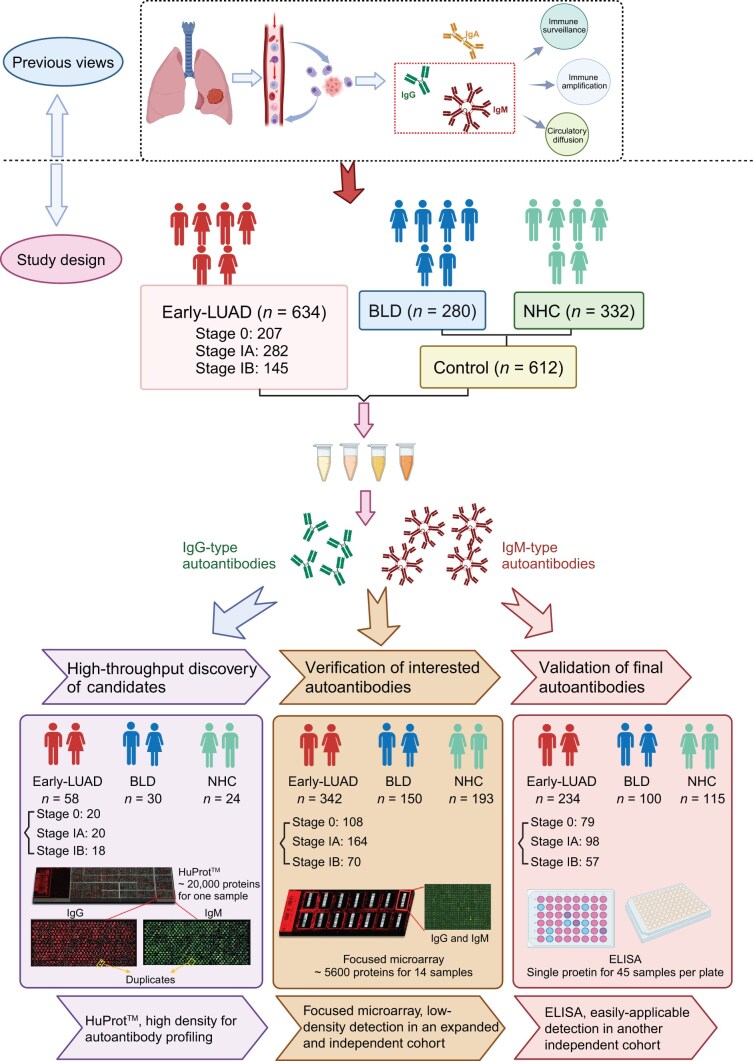
Flowchart of the study Early-LUAD, early-stage lung adenocarcinoma; BLD, benign lung disease; NHC, normal healthy control; HuProt^TM^, Human Proteome Microarray; ELISA, enzyme-linked immunosorbent assay.

This study employed a multi-step approach to identify novel IgG and IgM autoantibodies for Early-LUAD diagnosis. Plasma samples were obtained from 1246 individuals, comprising 634 patients with Early-LUAD, 280 patients with BLD, and 332 normal healthy controls (NHCs). Then, HuProt^TM^ discovery, focused microarray verification, and ELISA validation were conducted sequentially on three independent cohorts of 112, 685, and 449 individuals, respectively (**Figure 1**). Participants in all groups across the three cohorts were matched by sex and age. The average age of patients with Early-LUAD was approximately 55 years, with a male-to-female ratio close to 1:1 (**Table 1**).

**Table 1:** Clinical characteristics of study subjects

	**Discovery by HuProt^TM^ (*n* = 112)**				Verification by focused microarray (*n* = 685)				Validation by ELISA (*n* = 449)			
	Early-LUAD	BLD	NHC	*P* value	Early-LUAD	BLD	NHC	*P* value	Early-LUAD	BLD	NHC	*P* value
	(*n* = 58)	(*n* = 30)	(*n* = 24)		(*n* = 342)	(*n* = 150)	(*n* = 193)		(*n* = 234)	(*n* = 100)	(*n* = 115)	
**Age**												
Mean ± SD (year)	56.7 ± 8.0	57.5 ± 8.6	58.6 ± 8.3	0.63	55.5 ± 10.2	55.3 ± 8.9	55.8 ± 6.7	0.88	55.2 ± 10.2	56.3 ± 11.5	55.1 ± 5.7	0.57
**Gender**												
Male	25	15	12	0.77	164	76	109	0.18	119	58	69	0.22
Female	33	15	12		178	74	84		115	42	46	
**IMD**												
< 3 cm	50	28		0.48	293	103		0.31	201	64		0.83
≥ 3 cm	8	2			49	13			33	9		
NA	0	0			0	34			0	27		
**PMD**												
< 3 cm	49	28		0.32	292	103		0.29	201	7		0.95
≥ 3 cm	9	2			49	15			33	65		
NA	0	0			1	32			0	28		
**Lesion number**												
Single	49	28		0.32	280	106		0.17	189	68		0.02
Multiple	9	2			62	14			45	6		
NA	0	0			0	30			0	26		
**Smoke**												
Yes	12	14		0.01	76	38		0.05	54	22		0.39
No	46	16			266	82			180	52		
NA	0	0			0	30			0	26		
**Alcohol**												
Yes	9	13		< 0.01	56	30		0.04	49	23		0.07
No	49	17			286	90			185	51		
NA	0	0			0	30			0	26		
**Stage**												
Stage 0	20				108				79			
Stage IA	20				164				98			
Stage IB	18				70				57			

*Note:* HuProt^TM^, Human Proteome Microarray; ELISA, enzyme-linked immunosorbent assay; Early-LUAD, early-stage lung adenocarcinoma; BLD, benign lung disease; NHC, normal healthy control; IMD, imaging maximum diameter; PMD, pathological maximum diameter; SD, standard deviation; NA, not available.

### Discovery of highly specific IgG and IgM autoantibodies with distinct profiles between Early-LUAD and controls

**Figure 2:**
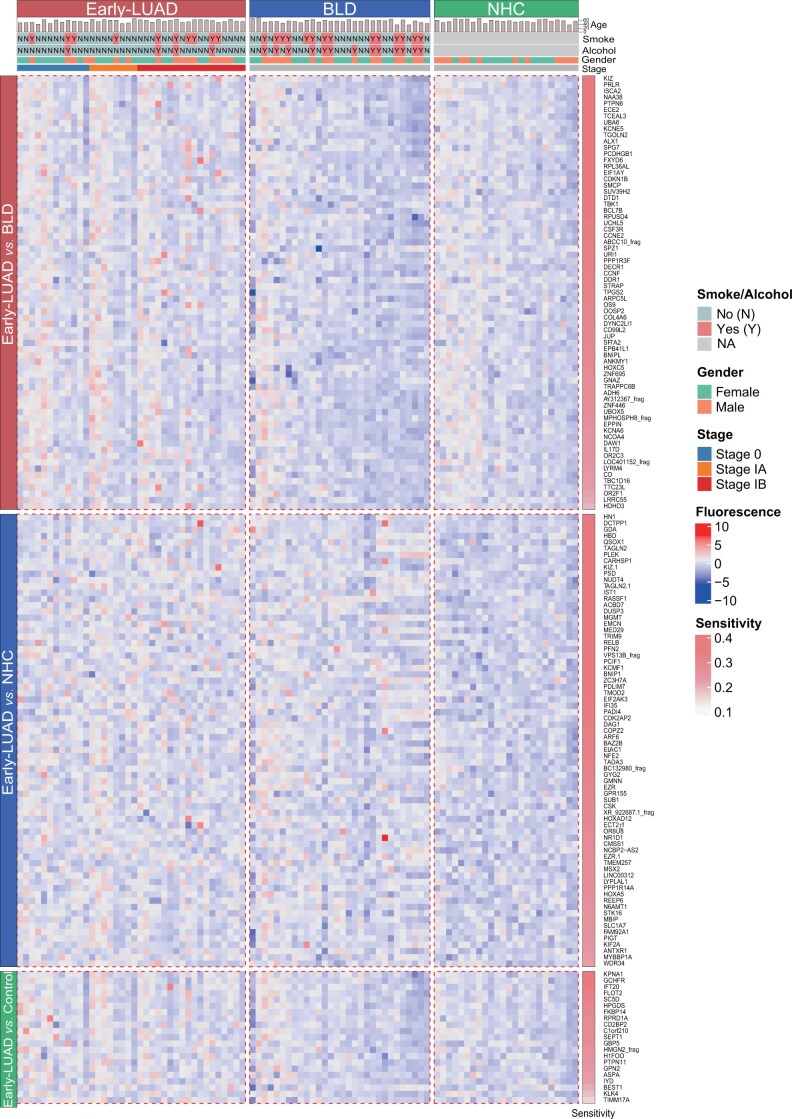
Profiling of significant IgM autoantibodies between Early-LUAD and BLD/NHC/Control in the HuProt^TM^ screening The heatmap displays a distinct distribution of significant IgM autoantibodies between Early-LUAD and BLD/NHC/Control, with generally higher levels of IgM observed in Early-LUAD. All values are normalized. The plots on the top display the data on age, sex, smoking, and alcohol consumption for each group, while the bar chart on the right side shows the sensitivity of each autoantibody. Control indicates the BLD+NHC group.

After normalization and background noise correction (Figure S1A and B), quality control confirmed high reproducibility, with a strong correlation of 0.97 between parallel duplicates for IgG and IgM detection in the HuProt^TM^ screening (Figure S1C and D). This discovery included samples from 58 patients with Early-LUAD, 30 patients with BLD, and 24 NHCs. Differential analysis revealed 229 IgG and 162 IgM candidates with significantly higher levels in the Early-LUAD group compared to the BLD/NHC/Control (BLD+NHC) group (Table S1). The sensitivity of these significant IgG autoantibodies ranged from 10.34% to 34.48%, with specificity exceeding 83.3%. Similarly, IgM autoantibodies demonstrated a sensitivity of 7.89%–36.84% and specificity of approximately 90% (Table S1). Normalized profiling of significant IgG and IgM autoantibodies across the three groups revealed a concentrated distribution of higher intensities in Early-LUAD (**Figure 2**, Figure S2).

A total of 417 IgG/IgM candidate autoantibodies were selected for focused microarray verification: 391 newly identified significantly differential autoantibodies [fold change (FC) > 1.0 and sensitivity > 7.5%] and 26 previously reported autoantibodies (Table S1).

### Focused microarray further verified 55 significantly elevated autoantibodies against 32 TAAs in Early-LUAD

**Figure 3:**
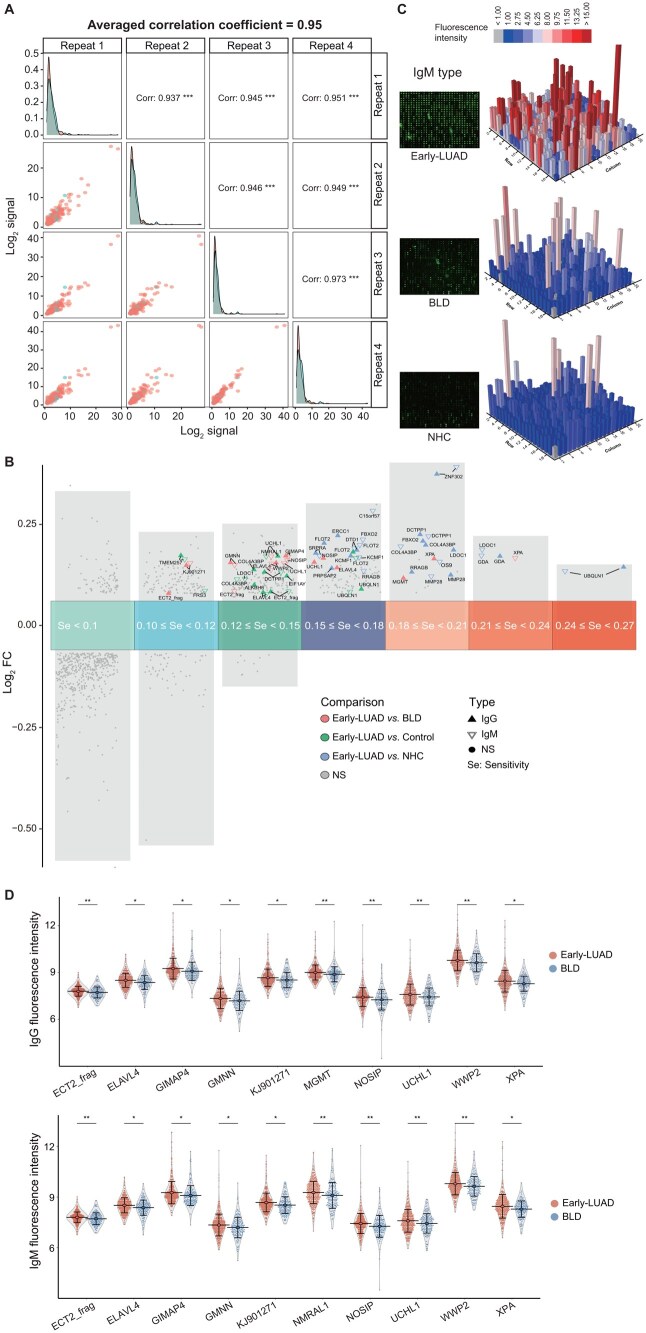
Verification of autoantibodies by focused microarray **A**. Repeated detection of pooled samples displayed high reproducibility, with an averaged correlation coefficient of 0.95. Pooled samples comprised randomly selected samples from the Early-LUAD, BLD, and NHC groups (10 samples in each group). ***, *P* < 0.001 (*t*-test for Pearson correlation coefficients). **B**. The top 10/top 15 IgG and IgM autoantibodies with the most significant elevation in Early-LUAD compared to BLD/NHC/Control are displayed. These most significant autoantibodies are ranked based on their FC on the vertical axis and sensitivity on the horizontal axis. **C**. A descending trend in the signal distribution of IgM autoantibodies across three representative samples from the Early-LUAD, BLD, and NHC groups, respectively. The images on the left visually depict the functionality of the focused microarray, while the 3D bar plots on the right show the distribution of normalized fluorescence intensities. **D**. IgG and IgM types of autoantibodies showed significantly higher levels in Early-LUAD compared to BLD. *, *P* < 0.05; **, *P* < 0.01 (Welch’s *t*-test). FC, fold change; NS, not significant.

Following the discovery, a custom-designed focused microarray was fabricated to contain the proteins targeted by the 417 IgG/IgM autoantibodies mentioned above. The focused microarray enabled the evaluation of 342 Early-LUAD, 150 BLD, and 193 NHC samples, with a capacity to detect 14 samples per assay. To assess inter-assay repeatability after normalization (Figure S3A and B), we applied representative mixed samples (pooled from 10 randomly chosen samples from each group) in parallel detection. This analysis yielded a high average correlation coefficient of 0.95, demonstrating good inter-assay repeatability (**Figure 3**A). Consistent results were obtained upon duplicate analyses, further supporting good intra-assay repeatability (Figure S3C). Additionally, we repeated the detection of bovine serum albumin (BSA) three times, and observed that the signal distribution remained consistent at approximately 1.0, indicating minimal non-specific binding (Figure S3D).

In the verification cohort, 32 TAAs were identified to be targeted by the top 10/top 15 most significant IgG and IgM autoantibodies in each comparison group (total number = 55). Selection criteria included FC and sensitivity values in each comparison group (Figure 3B; Table S2). Notably, these verified autoantibodies maintained high specificity, exceeding 90% for the detection of Early-LUAD. Their sensitivities for Early-LUAD detection ranged from 11.1% to 26.6%, while the positive rates in the BLD/NHC/Control group remained below 10% (Table S2).

Representative images and corresponding signal distributions of IgM and IgG autoantibodies across the Early-LUAD, BLD, and NHC groups are presented in Figure 3C and Figure S3E, respectively. As illustrated, targeted autoantibodies in Early-LUAD exhibited significantly increased signal intensities compared to BLD/NHC. Additionally, the significant autoantibodies identified in Early-LUAD *vs.* BLD/NHC/Control are shown in Figure 3D and Figure S4.

### ELISA ultimately validated a 10-autoantibody panel for diagnosing Early-LUAD

After excluding the TAAs with synthesis failures or insufficient purification yields, 15 recombinant proteins of the 32 TAAs were validated by ELISA in an independent cohort. This cohort comprised 234 patients with Early-LUAD, 100 patients with BLD, and 115 NHCs. A high correlation between repeated samples across different batches, low intra-batch variability, and consistent performance of negative and positive controls collectively confirmed the reliability of ELISA detection in this study (Figure S5A–C).

**Figure 4:**
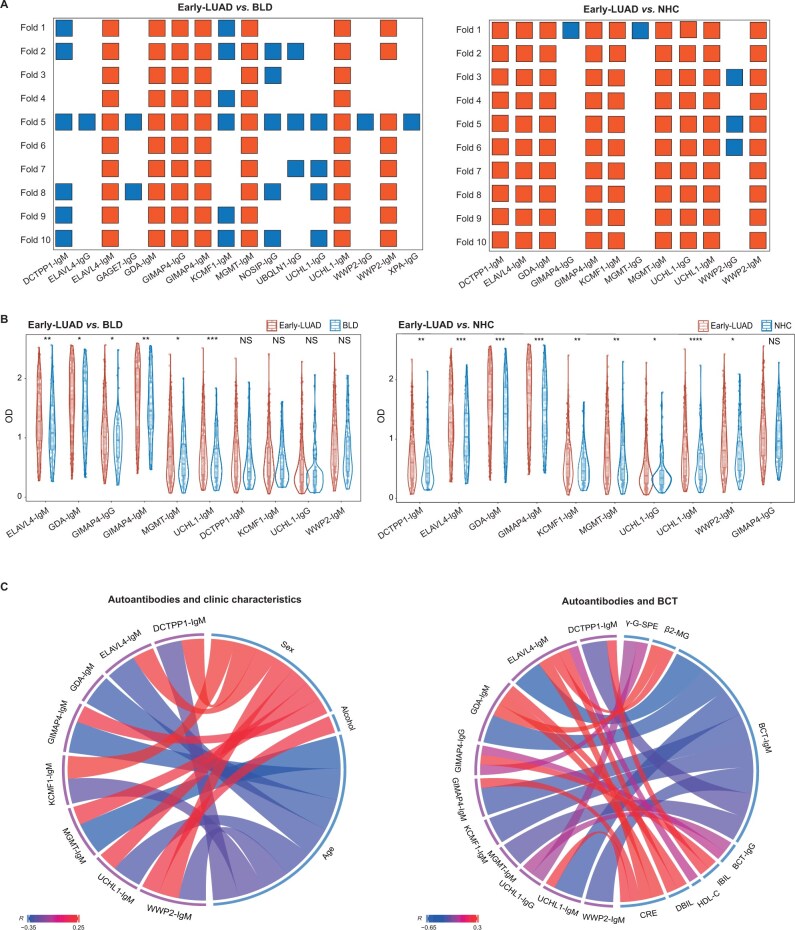
Validation of the 10-autoantibody panel by ELISA **A**. Targeted autoantibodies with significance were filtered using 10-fold differential analyses between Early-LUAD and BLD/NHC. The ELISA data were randomly divided into 10 folds. Red squares represent significant autoantibodies identified by all differential analyses (Welch’s *t*-test) in 10 folds, while blue squares represent non-statistically significant ones. **B**. Violin plots showing the higher levels of targeted autoantibodies in Early-LUAD compared to BLD (left) and NHC (right). **C**. Left: circular plot depicting the correlation between IgM autoantibodies and clinical characteristics (age, sex, and alcohol consumption). Right: circular plot showing the correlation between IgG/IgM autoantibodies and BCTs. Only statistically significant correlations (*P* < 0.05) are shown. The chords linking the autoantibodies and clinical characteristics/BCTs represent their correlations, with red indicating a positive association and blue indicating a negative association. The chord color reflects the correlation coefficient (*R*), with darker shades indicating stronger positive or negative correlations. *, *P* < 0.05; **, *P* < 0.01; ***, *P* < 0.001; NS, not significant (Welch’s *t*-test). OD, optical density; BCT, blood biochemical test; CRE, creatinine; DBIL, direct bilirubin; IBIL, indirect bilirubin; β2-MG, β2-microglobulin; γ-G-SPE, γ globulin in serum protein electrophoresis; HDL-C, high-density lipoprotein cholesterol.

To ensure the robustness of autoantibody selection, a 10-fold cross-validation approach was employed for differential analysis. Only autoantibodies that consistently demonstrated statistical significance across all 10 folds were selected for further analysis. After filtering duplicated autoantibodies, 10 unique autoantibodies were identified from Early-LUAD *vs.* BLD (*n* = 6) or Early-LUAD *vs.* NHC (*n* = 9). These 10 autoantibodies included ELAVL4-IgM, GDA-IgM, GIMAP4-IgM, GIMAP4-IgG, MGMT-IgM, UCHL1-IgM, DCTPP1-IgM, KCMF1-IgM, UCHL1-IgG, and WWP2-IgM (**Figure 4**A). This novel panel comprised commercially available UCHL1-IgG as well as newly identified IgG/IgM autoantibodies, exhibiting significantly higher levels in Early-LUAD compared to BLD/NHC controls (Figure 4B). The TAAs targeted by the newly identified autoantibodies displayed a close correlation with the tumorigenesis of lung cancer (Figure S6; Table S3).

The correlations between the ten autoantibodies and the clinical characteristics (age, sex, and alcohol consumption) or blood biochemical tests (BCTs) were investigated (Figure 4C). The eight IgM autoantibodies exhibited statistically significant negative correlations with age and positive correlations with sex or alcohol consumption. The eight IgM autoantibodies also displayed significant negative correlations with serum IgM levels (BCT-IgM). In contrast, the two IgG autoantibodies were positively correlated with serum IgG levels (BCT-IgG), suggesting distinct immunological roles of IgG and IgM antibodies in this context. Additionally, several autoantibodies demonstrated significant positive correlations with specific BCT parameters, including creatinine (CRE), direct bilirubin (DBIL), indirect bilirubin (IBIL), high-density lipoprotein cholesterol (HDL-C), γ globulin in serum protein electrophoresis (γ-G-SPE), and β2-microglobulin (β2-MG). No significant correlation was observed between the ten autoantibodies and the blood routine tests (data not shown).

### The 10-autoantibody panel demonstrated improved sensitivity for diagnosing Early-LUAD

**Figure 5:**
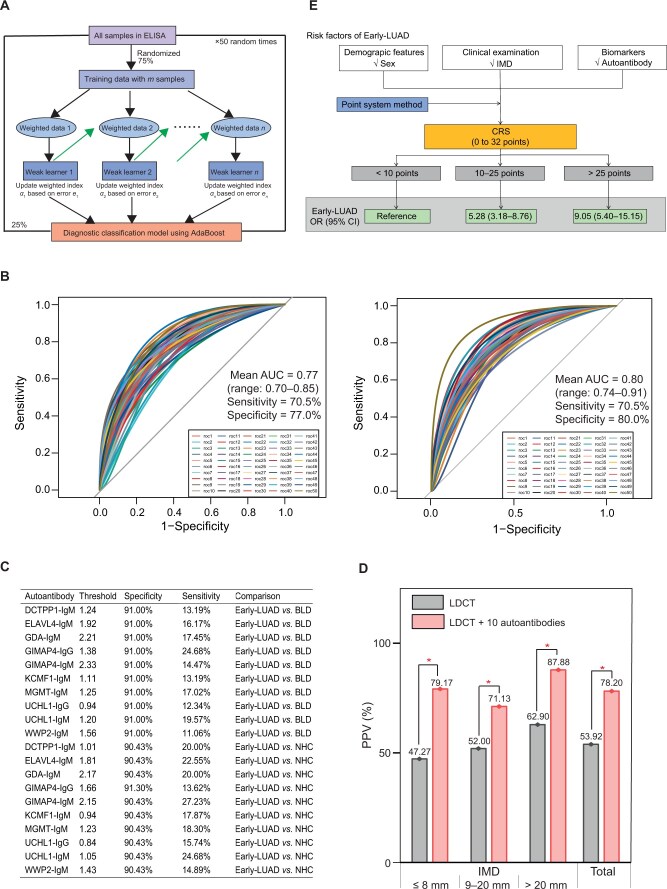
Classifier model and CRS based on the 10-autoantibody panel **A**. Flowchart of the classifier model utilizing AdaBoost machine learning. Model training was conducted with 75% of random samples from the ELISA cohort, while the remaining samples were used for the validation. The training–validation procedure of AdaBoost was repeated randomly 50 times for stable evaluation. **B**. ROC curves of 50-time models were generated, and the averaged AUC was calculated to distinguish Early-LUAD from BLD (left) or NHC (right). **C**. Sensitivities and specificities for individual autoantibodies. **D**. Combination of the 10-autoantibody panel with LDCT significantly enhanced the PPVs for differentiating lung nodules of varying IMDs. **E**. CRS that integrated sex, IMD, and the 10-autoantibody panel was established to assess the risk of Early-LUAD. *, *P* < 0.05 (chi-square test). ROC, receiver operation characteristic; AUC, area under the curve; PPV, positive predictive value; LDCT, low-dose computed tomography; IMD, imaging maximum diameter; OR, odds ratio; CI, confidence interval.

To assess the collective diagnostic potential of the 10 identified autoantibodies, we employed an AdaBoost machine learning model on the ELISA data. We randomly divided the ELISA cohort into training and validation sets with a ratio of 3:1. To mitigate overfitting and enhance generalizability, we employed a 50-iteration, 10-fold cross-validation approach within the training set, to generate 500 AdaBoost models (**Figure 5**A).

Evaluation of the validation set revealed that the area under the curve (AUC) ranged from 0.70 to 0.85 (mean: 0.77) or from 0.74 to 0.91 (mean: 0.80) for differentiating Early-LUAD from BLD or NHC (Figure 5B). Compared to individual autoantibodies, which exhibited sensitivities of 11.06%–24.68% with high specificity (> 90%), the 10-autoantibody panel achieved an improved sensitivity of 70.5% for Early-LUAD diagnosis, albeit with a trade-off in specificity (77.0% for distinguishing from BLD and 80.0% for NHC) (Figure 5B and C).

Furthermore, we evaluated the PPV of the 10-autoantibody panel in combination with LDCT for differentiating lung nodules with different imaging maximum diameters (IMDs) reported on LDCT scans. Compared to using LDCT alone, the combined approach significantly improved PPVs from 47.27% to 79.17% for nodules with IMD ≤ 8 mm, from 52.00% to 71.13% for nodules with 9 mm ≤ IMD ≤ 20 mm, and from 62.90% to 87.88% for nodules with IMD > 20 mm (Early-LUAD *vs.* BLD), respectively (Figure 5D).

### The combined risk score based on the 10-autoantibody panel facilitated the assessment of Early-LUAD risk

Building upon the concept of “point system” used in assessing coronary heart disease risk, we established a combined risk score (CRS) specifically for Early-LUAD. This score incorporated significant factors identified through β coefficients, including sex, IMD, and the dichotomized results of the 10-autoantibody panel (positive or negative). With the reference group defined as male, IMD ≤ 8 mm, and a negative 10-autoantibody panel result, the CRSs ranged from 0 to 32 points, with higher scores indicating an increased risk of Early-LUAD. Subsequently, the Early-LUAD and BLD groups were categorized based on their CRSs: < 10 points, 10–25 points, and > 25 points. Compared to patients with CRS < 10, individuals with 10 ≤ CRS ≤ 25 and CRS > 25 exhibited significantly elevated risks of Early-LUAD, with odds ratios (ORs) of 5.28 [95% confidence interval (CI): 3.18–8.76] and 9.05 (95% CI: 5.40–15.15), respectively (Figure 5E).

## Discussion

The success of autoantibody ELISA kits in lung cancer diagnosis underscores the importance of *de novo* discovery of novel autoantibodies to enhance early-stage detection. This study focused on patients with very early-stage (stage 0–I) LUAD. We employed a strategy that involved high-throughput microarray screening, followed by focused microarray and ELISA validation, to detect both IgG and IgM autoantibodies across three independent cohorts. We identified autoantibodies against two previously reported TAAs (UCHL1 and ELAVL4) as well as six novel TAAs, forming a new 10-autoantibody panel with improved sensitivity compared to previous studies. Our results demonstrated that the novel IgG–IgM panel combined with LDCT significantly enhanced the PPV for determining Early-LUAD compared to LDCT alone. Furthermore, the CRS provided an intuitive interpretation of the 10 autoantibodies with clinical diagnostic potential.

Since EarlyCDT^®^-Lung pioneered diagnostic autoantibody panels for lung cancer [10,31], the first Chinese panel, CancerProbe, has emerged with high similarity to EarlyCDT^®^-Lung. However, it lacks specific development tailored to the genetic background of Chinese population [13]. Both panels, comprised solely of IgG autoantibodies, demonstrated high specificity (> 90%) but limited sensitivity (30%–40% and 59%–62%) for stage I–II lung cancer diagnosis. Notably, these studies included a relatively small sample size of early-stage patients (*n* = 136 and *n* = 336). Subsequent studies explored the two panels’ performance across various clinical settings, revealing significant regional variations in sensitivity (30%–92.2%) and specificity (79.5%–95.2%) [15,32–34].

A key limitation of these earlier panels lies in the selection of IgG autoantibodies based solely on their TTAs’ functional relevance to cancer. However, the widespread presence of these TAAs across different cancers reduces the specificity of tumor origin inference, necessitating the direct screening of novel autoantibodies from patients with early-stage lung cancer in larger sample sizes. Utilizing HuProt^TM^, Pan et al. identified novel autoantibodies with enhanced sensitivities of 50% for the IgG panel (against p53, HRas, and ETHE1) and 73.5% for the combined IgG–IgA panel (against BCL7A, TRIM33, MTERF4, CTAG1A, DDX4, and MAGEC2), while maintaining a specificity of over 85% for early lung cancer detection [23,35]. Additionally, low-throughput targeted microarray (154 cancer driver genes) has also proven beneficial for discovering novel IgG autoantibodies (against p53, NPM1, FGFR2, PIK3CA, GNA11, HIST1H3B, and TSC1) (sensitivity = 94.4%, specificity = 82.7%) and IgM autoantibodies (against TSHR, ERBB2, survivin, PIK3CA, and JAK2) (sensitivity = 39.1%, specificity = 95.18%) [30,36]. While these studies are crucial for expanding the repertoire of new autoantibodies and exploring the potential of combining different antibody types, most lacked BLD control during screening, leaving the capacity to differentiate the nature of lung nodules uncertain. Moreover, the limited sample sizes [*n* = 9 in the screening phase [35]; *n* = 115 [36] and *n* = 83 [30] in the early stages (I–II)] may restrict the generality of the findings. Furthermore, these studies did not explore the ability of autoantibodies combined with LDCT for differentiating lung nodules.

Different types of antibodies reflect varying immune time windows and characteristics. While IgG and IgA have been the primary focus of previous studies, limited research suggests that IgM autoantibodies may be particularly informative. One study demonstrated that individual PD-1/PD-L1-IgM autoantibody was correlated with an increased risk of lung cancer, though only within age- or sex-stratified subgroups [21]. The aforementioned five-IgM-autoantibody panel also suggests the potential for IgM in early lung cancer detection [30]. Our study suggests that combining IgG, the most abundant immunoglobulin class, with IgM, the best indicator of early humoral response, could improve diagnostic sensitivity for Early-LUAD to 70.5%, while maintaining moderate specificities of 77.0% and 80.0% compared to BLD and NHC, respectively. Utilizing a large cohort of LUAD patients at truly early stage (stage 0–I, *n* = 634) and BLD control setting, our 10-autoantibody panel, identified through comprehensive screening and a reliable two-phase approach, demonstrates non-inferior diagnostic performance compared to previously reported IgG–IgA autoantibody panels. However, due to variations in study design, sample size, and control settings, direct comparisons of diagnostic accuracy require cautious interpretation. To the best of our knowledge, this study represents the first large-scale *de novo* investigation to identify IgG and IgM autoantibodies concurrently in the Chinese population. Notably, eight of the ten identified autoantibodies belong to the IgM class, potentially signifying their role in early immune surveillance, given the well-established temporal precedence of the IgM response.

Incorporating both established and newly identified autoantibodies may enhance the potential for diagnosing lung cancer. UCHL1 and ELAVL4, the “old” TAAs, are well-recognized biomarkers involved in deubiquitination and RNA translation processes, both of which contribute to tumor cell proliferation and survival [37–39]. UCHL1 participates in the early transformation and tumorigenesis of lung epithelial cells [40], and serum ELAVL4 levels are significantly higher in patients with lung cancer, indicating their diagnostic relevance [41].

The “new” autoantibodies in our panel target TAAs like DCTPP1, GIMAP4, WWP2, MGMT, KCMF1, and GDA, which have demonstrated links to lung cancer pathogenesis. DCTPP1, a nucleoside triphosphate catabolizing enzyme, has been implicated in cancer development through nuclear accumulation, promotion of cancer stemness, and enhancement of cell proliferation [42–44]. GIMAP4, a GTP-binding superfamily member, collaborates with caspases in programmed cell death and accelerates T-cell apoptosis [45–47], with its expression potentially impacting survival in lung cancer [48,49]. Elevated WWP2 has been linked to the development of LUAD [50], and the STAT6-WWP2-p27 axis is known to regulate cell proliferation, cell cycle progression, and apoptosis in lung cancer [51]. Moreover, the role of MGMT in DNA repair and its oncogenic implications due to promoter methylation in lung cancer are well-established [52–54]. KCMF1 and GDA, not previously reported in LUAD, have emerged as potential cancer biomarkers. KCMF1, a potassium channel regulatory factor, promotes the development of pancreatic cancer by modulating MAPK levels and driving cell proliferation, and it is also elevated in gastric cancer via FGF2 signaling pathway [55,56]. Although GDA is rarely explored in cancer, its presence in cells at the squamocolumnar junction, a known site of origin for cervical cancer, suggests a potential link to cancer [57]. These findings collectively suggest that our autoantibodies may target key biological changes underlying early-stage lung cancer. The documented associations between these TAAs and lung cancer pathogenesis enhance the validity of our platform in discovering novel and clinically relevant cancer biomarkers.

Our study has several limitations. First, the single-center, case-control design may introduce sample selection bias, underscoring the need of a multicenter study with a larger sample size for robust validation. Second, the clinical performance of the 10-autoantibody panel was evaluated only in LUAD, necessitating further investigation to assess its applicability for diagnosing other histological subtypes of lung cancer. Furthermore, since this is a cross-sectional study, a longitudinal cohort study is required to validate the predictive role of the 10 autoantibodies and the CRS system in lung cancer development.

Our study employed high-throughput discovery followed by stepwise validation to identify a novel IgG–IgM autoantibody panel. This panel demonstrates promising potential for early LUAD detection, particularly in enhancing the differentiation between malignant and benign nodules when used in conjunction with LDCT.

## Materials and methods

### Patients and NHCs

Plasma samples were collected sequentially from 1246 individuals between December 2019 and March 2021. As confirmed by surgical pathology, this study included 634 patients with Early-LUAD at stages 0, IA, and IB. Staging was conducted according to the 8th edition of the American Joint Committee on Cancer (AJCC)/Union for International Cancer Control (UICC) Tumor-Node-Metastasis (TNM) Staging System.

Additionally, 280 patients were histopathologically diagnosed as BLD with benign pulmonary nodules, such as pulmonary tuberculosis, suspected tuberculous nodules, organizing pneumonia, hamartoma, pulmonary sclerosing pneumocytoma, and inflammatory pseudotumor. Furthermore, we included 332 NHCs with no prior history of malignant tumors and no suspicious findings on serial imaging or laboratory examinations during their annual routine cancer screening.

### Plasma sample collection

Blood samples were obtained from patients with LUAD and BLD during initial diagnosis and prior to any surgical intervention. NHC samples were collected during routine physical examinations. Whole blood samples underwent immediate centrifugation at 3000 r/min for 10 min after collection. The supernatant was immediately stored at –80°C for subsequent analysis.

### High-throughput screening by HuProt^TM^

The high-throughput protein microarray HuProt^TM^ v4.0 was obtained from CDI Laboratories (Catalog No. CDIHP-004, Baltimore, MD). The protein library of HuProt^TM^ is derived from publicly available Open Reading Frames (ORFs). The validated expression plasmids are transferred into the *Saccharomyces cerevisiae* eukaryotic expression system to generate recombinant proteins with GST tags [58]. HuProt^TM^ v4.0 covers over 81% of the human proteome, with each protein printed in duplicate spots across 20 blocks for efficient reaction with a single plasma sample. The HuProt^TM^ screening procedures followed previously described protocols [59,60]. Briefly, microarrays stored at –80°C were equilibrated at room temperature for 20 min and then blocked with 5% BSA (5 ml) for 2 h. After removing the BSA, the microarrays were incubated with a diluted plasma sample (1:1000 dilution in 5% BSA, 5 ml) for 1 h at room temperature. Subsequently, the microarrays were washed three times for 10 min each with 0.1% phosphate buffered saline with Tween (PBST). Next, the microarrays were incubated with a pre-diluted secondary antibody mixture containing fluorescently labeled Alexa Fluor 647 goat anti-human IgG (Catalog No. 109-605-088, Jackson ImmunoResearch, West Grove, PA) and AcalephFluor532 goat anti-human IgM (Catalog No. CSA3307, Cohesion Biosciences, London, UK) at a 1:1000 dilution in a dark environment. After thoroughly washing with PBST, the microarrays were dried and scanned using a GenePix 4300A scanner (Molecular Devices, Sunnyvale, CA) at wavelengths of 635 nm (IgG) and 532 nm (IgM). The scanning parameters were set to a photomultiplier tube (PMT) gain of 700 and a scanning intensity power of 100. GenePix Pro software (v7.0) was used to extract foreground (F) and background (B) median fluorescence intensities. Each microarray underwent manual calibration by a designated individual for signal extraction, followed by a review by a second person. In cases of discrepancies, a third person ensured accuracy and consistency.

### Focused microarray verification

The low-density focused microarray was obtained from CDI Laboratories (Catalog No. CDIFA-U24L), and comprised two columns and seven rows of 14 blocks, with each block containing screened target proteins printed in duplicate spots, allowing for simultaneous testing with 14 plasma samples. The core procedures were similar to those of the HuProt^TM^ microarray experiment, except for the use of 3% BSA as the blocking buffer.

### ELISA validation of diagnostic autoantibodies

Recombinant proteins with GST tags (Catalog No. Ubio2021003, CDI Laboratories) were coated onto 96-well plates to detect the targeted autoantibodies, following established protocols [59]. Briefly, each well was coated with 50 ng of recombinant protein overnight at 4°C. After washing with 0.1% PBST three times, the plates were blocked with 50 μl of 5% skimmed milk for 2 h at room temperature. Plasma samples diluted 1:100 in 5% skimmed milk were incubated with the immobilized proteins for 1 h at 37°C. Positive controls (anti-GST antibody; Catalog No. ab19256, Abcam, Cambridge, UK) and blank controls (skimmed milk) were incubated on each plate. After washing with 0.1% PBST five times, 50 μl of goat anti-human IgG horseradish peroxidase (HRP) conjugated antibody (Catalog No. 109-035-088, Jackson ImmunoResearch) was added at a 1:20,000 dilution in phosphate buffered saline (PBS) for 1 h at 37°C in a dark environment. Following another five washes with 0.1% PBST, 75 μl of tetramethylbenzidine (TMB) substrate solution was added and allowed to react for 15 min at room temperature. The reaction was stopped by adding 75 μl of sulfuric acid solution. The optical density (OD) was measured at 450 nm using an automatic microplate reader (Multiskan GO, Thermo Fisher Scientific, Waltham, MA). The final OD value for each sample well was calculated by subtracting the blank control value.

### Data analysis

#### Microarray analysis

Fluorescent signals from both the HuProt^TM^ screening and focused microarray verification were normalized using R software (v4.2.1). The normalization process involved averaging “F635 Median” and “B635 Median” of duplicated spots and correcting for background intensities using the “normexp” along with a saddle-point approximation model in the Bioconductor package limma (v3.52.2). Background-corrected intensities were normalized using the cyclic loess method (Loess normalization) in the limma package. This Loess normalization ensured data comparability by restricting the values to a certain range (Figures S1A, S1B, S3A, and S3B). Based on the log2-transformed normalized data, differential analysis between groups was performed using a linear model with Bayes statistics. In the HuProt^TM^ screening, candidates were identified by comparing Early-LUAD to BLD/NHC/Control (BLD+NHC). Candidate autoantibodies with FC > 1.0, *P* < 0.05, and sensitivity > 10% for IgG and > 7.5% for IgM were selected. We supplemented the selection according to previously established methods [23,58,61]. For the focused microarray verification, we selected the top 10 or top 15 autoantibodies with the highest FCs and sensitivities, provided they were statistically significant (*P* < 0.05).

#### Quality control analysis

The reproducibility of microarrays was assessed by calculating the Pearson correlation coefficient *R* and the corresponding *P* value between duplicate spots and across microarrays. Non-specific binding in the focused microarrays was evaluated by concurrently testing BSA controls. The frequency distribution of BSA and test sample signal intensities was analyzed and fitted to a Gaussian distribution curve. The assay variability of ELISA detection was measured using the coefficient of variation (CV), defined as the standard deviation (σ) of replicate measurements divided by the average value (μ). A higher CV indicates greater assay variability and lower assay reproducibility.

#### Correlation analysis between autoantibodies and clinical characteristics/laboratory tests

Pearson correlation analysis was employed to assess the relationship between autoantibodies and continuous variables like age and blood laboratory tests. The Spearman rank correlation test was used to evaluate the correlation between autoantibodies and categorical variables like sex, stage, smoking status, and alcohol consumption.

#### AdaBoost machine learning model

We employed an AdaBoost machine learning model, which efficiently handles complex problems by boosting the accuracy of weak learners and combining them into a strong learner. To mitigate the potential bias due to unequal sample sizes, we employed the synthetic minority oversampling technique (SMOTE) algorithm (smotefamily package, v1.3.1) to oversample the group with a smaller sample size. After randomly splitting the data into training (75%) and validation (25%) sets using the caret package (v6.0-86), the “mfinal” parameter was fine-tuned through 10-fold cross-validation within the training set. The optimized model was evaluated using the validation set, and the training-validation process was randomly repeated 50 times to ensure robustness. Model performance was assessed using the average AUC from the receiver operating characteristic (ROC) analysis. Additionally, sensitivity and specificity were calculated using the confusion matrix.

#### Combined risk score

We developed a CRS based on a “point system” used in other diseases, such as coronary heart disease and upper gastrointestinal cancer [62,63]. The CRS was calculated through the following steps: (1) inclusion of significant variables (*i.e.*, sex, IMD measurement from LDCT, and a 10-autoantibody panel); (2) categorizing the risk factors into subgroups and setting dummy variables (value 0 or 1) for each subgroup, with W*_ij_* denoting the value for the *j*-th category of the *i*-th risk factor; (3) determining the base category for each risk factor and assigning 0 point (W*_i_*REF) for the base category in the scoring system; (4) calculating the distance of each category from the base category [*β_i_* (W_*ij*_ − W*_i_*REF)]; (5) defining the regression coefficient of sex (B) as a constant; (6) calculating the point number for each category of each risk factor [point number*_ij_* = *β_i_* (W*_ij_* − W_i_REF)/B]; (7) rounding the risk points to the nearest integers and calculating the CRS by summing individual risk points for each level of each risk factor. The final CRS ranged from 0 to 32 points, and we calculated the OR by dividing the CRS into three categories [< 10 (reference), 10–25, and > 25].

#### Other statistical analyses

We employed chi-square tests or Fisher’s exact tests, depending on sample size considerations, to compare the proportions of positive autoantibodies between groups. Welch’s *t*-test in R was used to assess differences in autoantibody levels between groups. *P* < 0.05 was considered statistically significant. When necessary due to multiple comparisons, we applied the Benjamini-Hochberg correction to adjust *P* values.

## Ethical statement

All plasma samples were obtained after routine clinical laboratory tests, with prior approval from the Ethics Review Committee of the Cancer Hospital Chinese Academy of Medical Sciences (Approval No. 19-019/1804). Since the samples were anonymized remnants from standard clinical procedures, a waiver of informed consent was obtained.

## Code availability

The code used in the study is deposited at GitHub (https://github.com/XHHLab/Autoantibody_LUAD). The code has also been submitted to BioCode at the National Genomics Data Center (NGDC), Beijing Institute of Genomics (BIG), Chinese Academy of Sciences (CAS) / China National Center for Bioinformation (CNCB) (BioCode: BT007912), which is publicly accessible at https://ngdc.cncb.ac.cn/biocode/tools/BT007912.

## Supplementary Material

qzae085_Supplementary_Data

## Data Availability

The raw data have been deposited in the OMIX [64] at the NGDC, BIG, CAS / CNCB (OMIX: OMIX006110), and are publicly accessible at https://ngdc.cncb.ac.cn/omix.

## CRediT author statement

**Rongrong Luo:** Data curation, Formal Analysis, Investigation, Methodology, Project administration, Validation, Visualization, Writing – original draft, Writing – review & editing. **Xiying Li:** Data curation, Resources, Writing – review & editing. **Ruyun Gao:** Investigation, Methodology, Validation, Writing – original draft, Writing – review & editing. **Mengwei Yang:** Investigation, Methodology, Validation. **Juan Cai:** Data curation, Resources. **Liyuan Dai:** Investigation, Methodology. **Ning Lou:** Investigation, Methodology. **Guangyu Fan:** Investigation, Methodology. **Haohua Zhu:** Investigation, Methodology. **Shasha Wang:** Investigation, Methodology. **Zhishang Zhang:** Project administration. **Le Tang:** Project administration. **Jiarui Yao:** Project administration. **Di Wu:** Project administration. **Yuankai Shi:** Conceptualization, Funding acquisition, Supervision, Writing – review & editing. **Xiaohong Han:** Conceptualization, Funding acquisition, Supervision, Writing – review & editing. All authors have read and approved the final manuscript.

## ORCID

0000-0003-0702-8471 (Rongrong Luo)

0000-0002-0766-3558 (Xiying Li)

0009-0004-3683-8734 (Ruyun Gao)

0009-0000-0652-6212 (Mengwei Yang)

0000-0001-8336-1415 (Juan Cai)

0009-0007-4848-3709 (Liyuan Dai)

0000-0003-0249-1192 (Ning Lou)

0000-0003-1561-0007 (Guangyu Fan)

0000-0002-7914-0289 (Haohua Zhu)

0000-0001-6262-0537 (Shasha Wang)

0000-0002-7876-0216 (Zhishang Zhang)

0000-0002-5192-3329 (Le Tang)

0000-0002-9835-395X (Jiarui Yao)

0009-0009-7526-8961 (Di Wu)

0000-0002-8685-6744 (Yuankai Shi)

0000-0001-9190-0167 (Xiaohong Han)
