# Supplementary material for: A Novel IgG–IgM Autoantibody Panel Enhances Detection of Early-stage Lung Adenocarcinoma from Benign Nodules
Source: Genomics Proteomics Bioinformatics. 2024 Dec 11;22(6):qzae085. doi: 10.1093/gpbjnl/qzae085 (PMC12032526; doi:10.1093/gpbjnl/qzae085)
Supplement: qzae085_Supplementary_Data [file qzae085_supplementary_data.zip › supplementary material captions.docx]

**Supplementary material**

**Figure S1 Normalization and quality control of the HuProt^TM^ discovery**

Distribution of non-normalized (**A**) and Loess-normalized (**B**) signal intensities from all samples in the HuProt^TM^ discovery set. **C.** Representative images of IgG and IgM autoantibody signals and their technical replicates (parallel spots) printed on the array. **D.** The strong correlation coefficient observed between signals from duplicate spots on the HuProt^TM^ microarray indicates high reproducibility of the assay.

**Figure S2 Profiling of significant IgG autoantibodies between Early-LUAD and BLD/NHC/Control in the HuProt^TM^ screening**

The heatmap displays a distinct distribution of significant IgG autoantibodies between Early-LUAD and BLD/NHC/Control, with generally higher levels of IgG observed in Early-LUAD. All autoantibody values have been normalized. The plot at the top displays the data on age, sex, smoking, and alcohol consumption for each group, while the bar chart on the right side shows the sensitivity of each autoantibody. Early-LUAD, early-stage lung adenocarcinoma; BLD, benign lung disease; NHC, normal healthy control; Control, benign lung disease and normal healthy control.

**Figure S3 Normalization, quality control, and significant differential autoantibodies identified in the focused array**

Distribution of non-normalized (**A**) and Loess-normalized (**B**) signal intensities from all samples in the focused array. **C.** The strong correlation between signals from duplicate spots (technical replicates) on the array indicates high reproducibility. **D.** Signal distribution of BSA indicates minimal non-specific binding. **E.** A descending trend in the signal distribution of IgG autoantibodies across three representative samples from Early-LUAD, BLD, and NHC. The image on the left side visually depicts the functionality of the focused array, alongside the 3D bar on the right showing the distribution of normalized fluorescence intensities. Early-LUAD, early-stage lung adenocarcinoma; BLD, benign lung disease; NHC, normal healthy control.

**Figure S4 Significant autoantibodies identified in the focused array**

Significant IgG and IgM autoantibodies identified by comparing Early-LUAD with NHC (**A**) and the combined Control (BLD+NHC) (**B**). Early-LUAD, early-stage lung adenocarcinoma; BLD, benign lung disease; NHC, normal healthy control; AAbs, autoantibodies.

**Figure S5 Quality control assessment of ELISA validation**

**A.** Coefficient of variation (CV) between duplicate samples for autoantibodies detected in ELISA. Low CV indicates good repeatability. **B.** Correlation between signals from the same samples measured at different time points indicates a good inter-plates reproducibility. **C.** The distribution of signal intensities for test samples between the PC and NC thresholds suggests stable measurement. PC, positive control; NC, negative control.

**Figure S6 Functional enrichment analysis of TAAs targeted by newly discovered autoantibodies**

**A.** Gene Ontology (GO) enrichment suggests that TAAs are associated with nucleotide metabolism process and function. **B.** Kyoto Encyclopedia of Genes and Genomes (KEGG) pathway enrichment indicates that the TAAs are significantly enriched in nucleotide metabolism pathways. TAAs, tumor-associated antigens.

**Table S1 Significant differential autoantibodies in the HuProt^TM^ discovery**

**Table S2 Candidate autoantibodies in the focused array**

**Table S3 The biological function of eight TAAs targeted by ELISA-validated autoantibodies**
