# Supplementary material for: A Novel IgG–IgM Autoantibody Panel Enhances Detection of Early-stage Lung Adenocarcinoma from Benign Nodules
Source: Genomics Proteomics Bioinformatics. 2024 Dec 11;22(6):qzae085. doi: 10.1093/gpbjnl/qzae085 (PMC12032526; doi:10.1093/gpbjnl/qzae085)
Supplement: qzae085_Supplementary_Data [file qzae085_supplementary_data.zip › Table S3.docx]

**Table S3 The biological function of eight TAAs targeted by ELISA-validated autoantibodies**

| **TAAs** | **Autoantibodies type** | **Protein name** | **ligand** | **Molecular function** | **Biological process** | **Cellular component** | **Access No.** |
| --- | --- | --- | --- | --- | --- | --- | --- |
| ELAVL4 | IgM | ELAV-like protein 4 |  | [RNA-binding](https://www.uniprot.org/keywords/KW-0694) | mRNA processing  mRNA splicing | Cell projection  Cytoplasm | P26378 |
| GDA | IgM | Guanine deaminase | [Metal-binding](https://www.uniprot.org/keywords/KW-0479)  [Zinc](https://www.uniprot.org/keywords/KW-0862) | [Hydrolase](https://www.uniprot.org/keywords/KW-0378) |  |  | Q9Y2T3 |
| GIMAP4 | IgM, IgG | GTPase IMAP family member 4 | [GTP-binding](https://www.uniprot.org/keywords/KW-0342)  [Nucleotide-binding](https://www.uniprot.org/keywords/KW-0547) |  |  | [Cytoplasm](https://www.uniprot.org/keywords/KW-0963) | Q9NUV9 |
| MGMT | IgM | Methylated-DNA--protein-cysteine methyltransferase | [Metal-binding](https://www.uniprot.org/keywords/KW-0479)  [Zinc](https://www.uniprot.org/keywords/KW-0862) | [DNA-binding](https://www.uniprot.org/keywords/KW-0238)  [Methyltransferase](https://www.uniprot.org/keywords/KW-0489)  [Transferase](https://www.uniprot.org/keywords/KW-0808) | DNA damage  DNA repair | [Nucleus](https://www.uniprot.org/keywords/KW-0539) | P16455 |
| UCHL1 | IgM, IgG | Ubiquitin carboxyl-terminal hydrolase isozyme L1 |  | [Hydrolase](https://www.uniprot.org/keywords/KW-0378)  [Protease](https://www.uniprot.org/keywords/KW-0645)  [Thiol protease](https://www.uniprot.org/keywords/KW-0788) | Ubl conjugation pathway | [Cytoplasm](https://www.uniprot.org/keywords/KW-0963)  [Endoplasmic reticulum](https://www.uniprot.org/keywords/KW-0256)  [Membrane](https://www.uniprot.org/keywords/KW-0472) | P09936 |
| DCTPP1 | IgM | dCTP pyrophosphatase 1 | [Magnesium](https://www.uniprot.org/keywords/KW-0460)  [Metal-binding](https://www.uniprot.org/keywords/KW-0479)  [Nucleotide-binding](https://www.uniprot.org/keywords/KW-0547) | [Hydrolase](https://www.uniprot.org/keywords/KW-0378) |  | [Cytoplasm](https://www.uniprot.org/keywords/KW-0963)  [Mitochondrion](https://www.uniprot.org/keywords/KW-0496)  [Nucleus](https://www.uniprot.org/keywords/KW-0539) | Q9H773 |
| KCMF1 | IgM | E3 ubiquitin-protein ligase KCMF1 | [Metal-binding](https://www.uniprot.org/keywords/KW-0479)  [Zinc](https://www.uniprot.org/keywords/KW-0862) | [Transferase](https://www.uniprot.org/keywords/KW-0808) | Ubl conjugation pathway |  | Q9P0J7 |
| WWP2 | IgM | NEDD4-like E3 ubiquitin-protein ligase WWP2 |  | [Transferase](https://www.uniprot.org/keywords/KW-0808) | Host-virus interaction  Ubl conjugation pathway | [Nucleus](https://www.uniprot.org/keywords/KW-0539) | O00308 |
