# Supplementary material for: A Novel IgG–IgM Autoantibody Panel Enhances Detection of Early-stage Lung Adenocarcinoma from Benign Nodules
Source: Genomics Proteomics Bioinformatics. 2024 Dec 11;22(6):qzae085. doi: 10.1093/gpbjnl/qzae085 (PMC12032526; doi:10.1093/gpbjnl/qzae085)
Supplement: qzae085_Supplementary_Data [file qzae085_supplementary_data.zip › Table S1.docx]

**Table S1 Significant differential autoantibodies in the HuProt^TM^ discovery**

| **ID** | **Name** | **Method** | **Comparison** | **Type** | **Log_2_FC** | **Sensitivity** | **Specificity** |
| --- | --- | --- | --- | --- | --- | --- | --- |
| JHU00140.P189A05 | IRAK4 | Loess | Early-LUAD *vs.* BLD | IgG | 1.559908 | 10.34% | 93.33% |
| JHU00140.P002G06 | IRAK4 | Loess | Early-LUAD *vs.* BLD | IgG | 1.455697 | 12.07% | 100.00% |
| JHU06997.P005C07 | TIPIN | Loess | Early-LUAD *vs.* BLD | IgG | 0.980206 | 13.79% | 93.33% |
| JHU06997.P066A12 | TIPIN | Loess | Early-LUAD *vs.* BLD | IgG | 0.815877 | 12.07% | 96.67% |
| JHU11437.P120D11 | C15orf57 | Loess | Early-LUAD *vs.* BLD | IgG | 0.773975 | 10.34% | 100.00% |
| JHU06997.P073A09 | TIPIN | Loess | Early-LUAD *vs.* BLD | IgG | 0.725533 | 12.07% | 96.67% |
| JHU00980.P011B02 | AP1AR | Loess | Early-LUAD *vs.* BLD | IgG | 0.690104 | 12.07% | 100.00% |
| JHU02539.P189G02 | BABAM1 | Loess | Early-LUAD *vs.* BLD | IgG | 0.672225 | 10.34% | 96.67% |
| JHU00997.P011B01 | NXPE3 | Loess | Early-LUAD *vs.* BLD | IgG | 0.602811 | 10.34% | 90.00% |
| JHU08293.P087H05 | FBXO2 | Loess | Early-LUAD *vs.* BLD | IgG | 0.575519 | 10.34% | 96.67% |
| Auto-antigen.P267A09 | SCL-70 | Loess | Early-LUAD *vs.* BLD | IgG | 0.571871 | 22.41% | 96.67% |
| JHU26236.P241D10 | JADE2 | Loess | Early-LUAD *vs.* BLD | IgG | 0.566492 | 12.07% | 93.33% |
| JHU00473.P005A02 | CNPY2 | Loess | Early-LUAD *vs.* BLD | IgG | 0.564606 | 13.79% | 93.33% |
| JHU13200.P138B09 | P3H4 | Loess | Early-LUAD *vs.* BLD | IgG | 0.556717 | 10.34% | 93.33% |
| JHU06315.P066H05 | PKIB | Loess | Early-LUAD *vs.* BLD | IgG | 0.540091 | 15.52% | 96.67% |
| JHU05837.P061E04 | TAF7L | Loess | Early-LUAD *vs.* BLD | IgG | 0.536835 | 10.34% | 96.67% |
| JHU24611.P244D01 | ZNF106 | Loess | Early-LUAD *vs.* BLD | IgG | 0.528342 | 13.79% | 90.00% |
| JHU25610.P243G09 | MAP1A | Loess | Early-LUAD *vs.* BLD | IgG | 0.523996 | 17.24% | 93.33% |
| JHU14292.P150A04 | CSTF2T | Loess | Early-LUAD *vs.* BLD | IgG | 0.522644 | 12.07% | 93.33% |
| JHU00309.P004D11 | CNOT2 | Loess | Early-LUAD *vs.* BLD | IgG | 0.52263 | 10.34% | 96.67% |
| JHU01783.P019A09 | MAGEB2 | Loess | Early-LUAD *vs.* BLD | IgG | 0.515344 | 13.79% | 93.33% |
| JHU02500.P027C01 | AKR1B1 | Loess | Early-LUAD *vs.* BLD | IgG | 0.515194 | 10.34% | 83.33% |
| JHU06426.P067C07 | UTP14A | Loess | Early-LUAD *vs.* BLD | IgG | 0.514947 | 13.79% | 100.00% |
| JHU02754.P004C07 | PDCD10 | Loess | Early-LUAD *vs.* BLD | IgG | 0.514652 | 10.34% | 93.33% |
| JHU00720.P008H11 | NEIL2 | Loess | Early-LUAD *vs.* BLD | IgG | 0.509074 | 17.24% | 96.67% |
| JHU01201.P013A09 | NOSIP | Loess | Early-LUAD *vs.* BLD | IgG | 0.495221 | 34.48% | 83.33% |
| JHU04026.P042B10 | TPD52L1 | Loess | Early-LUAD *vs.* BLD | IgG | 0.488297 | 10.34% | 93.33% |
| JHU18027.P223D07 | SFMBT2 | Loess | Early-LUAD *vs.* BLD | IgG | 0.487744 | 12.07% | 93.33% |
| JHU10279.P234B01 | BIN1 | Loess | Early-LUAD *vs.* BLD | IgG | 0.472099 | 12.07% | 96.67% |
| JHU18383.P211D07 | ME3 | Loess | Early-LUAD *vs.* BLD | IgG | 0.471843 | 10.34% | 93.33% |
| JHU00692.P008E05 | CCNE2 | Loess | Early-LUAD *vs.* BLD | IgG | 0.466775 | 20.69% | 86.67% |
| JHU10279.P188A08 | BIN1 | Loess | Early-LUAD *vs.* BLD | IgG | 0.462074 | 15.52% | 96.67% |
| JHU29554.P257B07 | ZGRF1 | Loess | Early-LUAD *vs.* BLD | IgG | 0.454761 | 13.79% | 96.67% |
| JHU07156.P075A05 | MAPK10 | Loess | Early-LUAD *vs.* BLD | IgG | 0.449495 | 15.52% | 100.00% |
| JHU02957.P031D10 | SPTB*vs.*frag | Loess | Early-LUAD *vs.* BLD | IgG | 0.444767 | 12.07% | 96.67% |
| JHU05061.P053C09 | PSEN2 | Loess | Early-LUAD *vs.* BLD | IgG | 0.439815 | 10.34% | 100.00% |
| JHU08491.P089F10 | RBM42 | Loess | Early-LUAD *vs.* BLD | IgG | 0.429928 | 15.52% | 96.67% |
| JHU07879.P083D11 | TYMSOS | Loess | Early-LUAD *vs.* BLD | IgG | 0.429738 | 13.79% | 100.00% |
| JHU08693.P091A05 | MAN2B1 | Loess | Early-LUAD *vs.* BLD | IgG | 0.422558 | 15.52% | 90.00% |
| JHU09085.P095D09 | P4HB | Loess | Early-LUAD *vs.* BLD | IgG | 0.415883 | 12.07% | 100.00% |
| JHU02923.P031B02 | ISY1 | Loess | Early-LUAD *vs.* BLD | IgG | 0.414169 | 10.34% | 96.67% |
| JHU05254.P055E01 | RFX3 | Loess | Early-LUAD *vs.* BLD | IgG | 0.400163 | 12.07% | 96.67% |
| JHU16058.P169G01 | AGTR1 | Loess | Early-LUAD *vs.* BLD | IgG | 0.40007 | 10.34% | 96.67% |
| JHU09882.P187H01 | XPA | Loess | Early-LUAD *vs.* BLD | IgG | 0.396973 | 13.79% | 100.00% |
| JHU14354.P229H11 | UTP6 | Loess | Early-LUAD *vs.* BLD | IgG | 0.39044 | 12.07% | 100.00% |
| JHU01007.P013B10 | HNRNPA0 | Loess | Early-LUAD *vs.* BLD | IgG | 0.388938 | 18.97% | 93.33% |
| JHU06268.P066A10 | KCMF1 | Loess | Early-LUAD *vs.* BLD | IgG | 0.387584 | 13.79% | 96.67% |
| JHU06975.P073B11 | PDE4DIP | Loess | Early-LUAD *vs.* BLD | IgG | 0.381337 | 12.07% | 100.00% |
| JHU07123.P075C07 | CYP2E1 | Loess | Early-LUAD *vs.* BLD | IgG | 0.38065 | 15.52% | 96.67% |
| JHU10740.P112D01 | LOC100507507 | Loess | Early-LUAD *vs.* BLD | IgG | 0.374613 | 22.41% | 86.67% |
| JHU09622.P187G04 | DAP | Loess | Early-LUAD *vs.* BLD | IgG | 0.371115 | 10.34% | 100.00% |
| JHU19479.P253D03 | GRK4 | Loess | Early-LUAD *vs.* BLD | IgG | 0.36863 | 10.34% | 96.67% |
| JHU08043.P084C03 | ZNF280B | Loess | Early-LUAD *vs.* BLD | IgG | 0.36565 | 15.52% | 93.33% |
| JHU07018.P074F01 | CDCA8 | Loess | Early-LUAD *vs.* BLD | IgG | 0.345413 | 10.34% | 100.00% |
| JHU02521.P027E08 | DDX39A | Loess | Early-LUAD *vs.* BLD | IgG | 0.344707 | 20.69% | 90.00% |
| JHU00967.P011B09 | ALKBH8 | Loess | Early-LUAD *vs.* BLD | IgG | 0.344429 | 10.34% | 90.00% |
| JHU12078.P127D01 | TTC39B | Loess | Early-LUAD *vs.* BLD | IgG | 0.343618 | 13.79% | 100.00% |
| JHU10603.P111D01 | LILRA5 | Loess | Early-LUAD *vs.* BLD | IgG | 0.342666 | 15.52% | 96.67% |
| JHU01415.P236A06 | RABL2B | Loess | Early-LUAD *vs.* BLD | IgG | 0.3281 | 12.07% | 100.00% |
| JHU07569.P079D06 | SHOC2 | Loess | Early-LUAD *vs.* BLD | IgG | 0.323139 | 13.79% | 93.33% |
| JHU11642.P122F04 | GAS2L1 | Loess | Early-LUAD *vs.* BLD | IgG | 0.318523 | 10.34% | 96.67% |
| JHU14170.P148C01 | ZNF574 | Loess | Early-LUAD *vs.* BLD | IgG | 0.317488 | 10.34% | 100.00% |
| JHU05751.P060D09 | TSGA13 | Loess | Early-LUAD *vs.* BLD | IgG | 0.312726 | 12.07% | 93.33% |
| JHU14208.P149F04 | IFT140 | Loess | Early-LUAD *vs.* BLD | IgG | 0.312273 | 10.34% | 93.33% |
| JHU06175.P065B06 | KJ903742 | Loess | Early-LUAD *vs.* BLD | IgG | 0.312209 | 10.34% | 96.67% |
| JHU15337.P161H11 | LGALS8 | Loess | Early-LUAD *vs.* BLD | IgG | 0.30318 | 10.34% | 100.00% |
| JHU06769.P071C11 | ZNF880 | Loess | Early-LUAD *vs.* BLD | IgG | 0.301634 | 10.34% | 100.00% |
| JHU07509.P079D08 | DYNC1I1 | Loess | Early-LUAD *vs.* BLD | IgG | 0.297208 | 15.52% | 100.00% |
| JHU05821.P061E07 | SEC31A | Loess | Early-LUAD *vs.* BLD | IgG | 0.29393 | 10.34% | 90.00% |
| JHU12126.P127B12 | PLA2R1 | Loess | Early-LUAD *vs.* BLD | IgG | 0.290636 | 12.07% | 96.67% |
| JHU08613.P090D04 | SENP6 | Loess | Early-LUAD *vs.* BLD | IgG | 0.288039 | 12.07% | 90.00% |
| JHU10608.P111E08 | LOC401052 | Loess | Early-LUAD *vs.* BLD | IgG | 0.276071 | 12.07% | 100.00% |
| JHU08395.P088C10 | CMTR1 | Loess | Early-LUAD *vs.* BLD | IgG | 0.271934 | 20.69% | 90.00% |
| JHU08638.P090E02 | XBP1 | Loess | Early-LUAD *vs.* BLD | IgG | 0.267379 | 10.34% | 100.00% |
| JHU06405.P067B01 | RPS24 | Loess | Early-LUAD *vs.* BLD | IgG | 0.257516 | 10.34% | 93.33% |
| JHU14068.P147D09 | XRCC2 | Loess | Early-LUAD *vs.* BLD | IgG | 0.252505 | 10.34% | 90.00% |
| JHU14043.P147D05 | PTPMT1 | Loess | Early-LUAD *vs.* BLD | IgG | 0.252295 | 12.07% | 93.33% |
| JHU11244.P118A12 | CAT | Loess | Early-LUAD *vs.* BLD | IgG | 0.246941 | 10.34% | 96.67% |
| JHU06743.P071C09 | DYNLRB2 | Loess | Early-LUAD *vs.* BLD | IgG | 0.239733 | 12.07% | 93.33% |
| JHU06911.P072F02 | KJ904395 | Loess | Early-LUAD *vs.* BLD | IgG | 0.222639 | 10.34% | 96.67% |
| JHU06608.P069A11 | SARS | Loess | Early-LUAD *vs.* BLD | IgG | 0.217043 | 20.69% | 90.00% |
| JHU04931.P052C04 | GIMAP4 | Supplement methods | Early-LUAD *vs.* NHC | IgG | 11.74965 | 18.97% | 91.67% |
| JHU04987.P052D05 | WWP2 | Supplement methods | Early-LUAD *vs.* NHC | IgG | 10.28567 | 17.24% | 91.67% |
| JHU03919.P041F05 | SHFM1 | Loess | Early-LUAD *vs.* NHC | IgG | 0.746905 | 15.52% | 100.00% |
| JHU04446.P047C09 | HDGFRP3 | Loess | Early-LUAD *vs.* NHC | IgG | 0.647661 | 25.86% | 95.83% |
| JHU08814.P092D10 | SPR | Loess | Early-LUAD *vs.* NHC | IgG | 0.642422 | 24.14% | 95.83% |
| JHU16547.P174D06 | PGK1 | Loess | Early-LUAD *vs.* NHC | IgG | 0.530994 | 24.14% | 100.00% |
| JHU03140.P236E07 | DNAJC27 | Loess | Early-LUAD *vs.* NHC | IgG | 0.527065 | 17.24% | 95.83% |
| JHU15810.P166C05 | MAGEB10 | Loess | Early-LUAD *vs.* NHC | IgG | 0.519457 | 10.34% | 100.00% |
| JHU07518.P234G09 | PYROXD1 | Loess | Early-LUAD *vs.* NHC | IgG | 0.515538 | 17.24% | 95.83% |
| JHU25415.P251H06 | LRRFIP1 | Loess | Early-LUAD *vs.* NHC | IgG | 0.51548 | 20.69% | 100.00% |
| JHU19451.P198F01 | AKT2 | Loess | Early-LUAD *vs.* NHC | IgG | 0.49468 | 12.07% | 100.00% |
| JHU13103.P137F10 | TFAP2E | Loess | Early-LUAD *vs.* NHC | IgG | 0.487 | 27.59% | 95.83% |
| JHU17488.P253D11 | SLC30A10 | Loess | Early-LUAD *vs.* NHC | IgG | 0.480663 | 17.24% | 100.00% |
| JHU29209.P254A12 | DBX1 | Loess | Early-LUAD *vs.* NHC | IgG | 0.45175 | 15.52% | 100.00% |
| JHU09253.P187F09 | EIF3F | Loess | Early-LUAD *vs.* NHC | IgG | 0.44968 | 17.24% | 91.67% |
| JHU02132.P229A11 | DTNA | Loess | Early-LUAD *vs.* NHC | IgG | 0.447512 | 13.79% | 95.83% |
| JHU06735.P071E04 | CNKSR3 | Loess | Early-LUAD *vs.* NHC | IgG | 0.440749 | 10.34% | 95.83% |
| JHU04981.P248F08 | TP53I3 | Loess | Early-LUAD *vs.* NHC | IgG | 0.433337 | 12.07% | 95.83% |
| JHU06169.P065E07 | CSNK1G2 | Loess | Early-LUAD *vs.* NHC | IgG | 0.426591 | 15.52% | 95.83% |
| JHU10892.P114H03 | LARP7 | Loess | Early-LUAD *vs.* NHC | IgG | 0.42635 | 18.97% | 100.00% |
| JHU04769.P050D06 | RPL3 | Loess | Early-LUAD *vs.* NHC | IgG | 0.425703 | 18.97% | 100.00% |
| JHU09615.P187G03 | C6orf201 | Loess | Early-LUAD *vs.* NHC | IgG | 0.423237 | 15.52% | 100.00% |
| JHU11728.P123F04 | CKAP2 | Loess | Early-LUAD *vs.* NHC | IgG | 0.408764 | 10.34% | 100.00% |
| JHU02916.P031F03 | GMDS | Loess | Early-LUAD *vs.* NHC | IgG | 0.402237 | 10.34% | 100.00% |
| JHU08182.P086E11 | DENND1C | Loess | Early-LUAD *vs.* NHC | IgG | 0.400642 | 17.24% | 100.00% |
| JHU18646.P215H02 | ADAMTS16 | Loess | Early-LUAD *vs.* NHC | IgG | 0.400357 | 15.52% | 100.00% |
| JHU08826.P092E10 | ZC3H10 | Loess | Early-LUAD *vs.* NHC | IgG | 0.397605 | 10.34% | 95.83% |
| JHU16619.P175F03 | ZNF524 | Loess | Early-LUAD *vs.* NHC | IgG | 0.391168 | 10.34% | 95.83% |
| JHU14748.P155D11 | GAB2 | Loess | Early-LUAD *vs.* NHC | IgG | 0.385249 | 12.07% | 100.00% |
| JHU05403.P057H09 | H2AFY | Loess | Early-LUAD *vs.* NHC | IgG | 0.385231 | 15.52% | 100.00% |
| JHU19558.P204G01 | PTPN20 | Loess | Early-LUAD *vs.* NHC | IgG | 0.383099 | 12.07% | 95.83% |
| JHU13028.P137G11 | C9orf114 | Loess | Early-LUAD *vs.* NHC | IgG | 0.379021 | 22.41% | 100.00% |
| JHU10078.P105G08 | ZNHIT1 | Loess | Early-LUAD *vs.* NHC | IgG | 0.366529 | 17.24% | 100.00% |
| JHU01811.P233H08 | SRSF12 | Loess | Early-LUAD *vs.* NHC | IgG | 0.363539 | 22.41% | 100.00% |
| JHU14028.P180D10 | NTHL1 | Loess | Early-LUAD *vs.* NHC | IgG | 0.361145 | 12.07% | 100.00% |
| JHU19695.P253D01 | Zar1 | Loess | Early-LUAD *vs.* NHC | IgG | 0.358766 | 17.24% | 100.00% |
| JHU08280.P087D06 | YBX3 | Loess | Early-LUAD *vs.* NHC | IgG | 0.358561 | 12.07% | 95.83% |
| JHU20355.P248B08 | GPBP1 | Loess | Early-LUAD *vs.* NHC | IgG | 0.357312 | 15.52% | 100.00% |
| JHU11329.P119H11 | ATE1 | Loess | Early-LUAD *vs.* NHC | IgG | 0.3552 | 15.52% | 100.00% |
| JHU07331.P080G01 | FXN | Loess | Early-LUAD *vs.* NHC | IgG | 0.348149 | 13.79% | 100.00% |
| JHU07950.P083D07 | SRPRA | Loess | Early-LUAD *vs.* NHC | IgG | 0.344365 | 17.24% | 100.00% |
| JHU06619.P069E09 | UPF3A | Loess | Early-LUAD *vs.* NHC | IgG | 0.33966 | 13.79% | 100.00% |
| JHU29796.P259G01 | GOLGA8F; GOLGA8G | Loess | Early-LUAD *vs.* NHC | IgG | 0.338675 | 15.52% | 100.00% |
| JHU15286.P160H06 | KJ901271 | Loess | Early-LUAD *vs.* NHC | IgG | 0.334604 | 15.52% | 100.00% |
| JHU14707.P154E09 | ZKSCAN4 | Loess | Early-LUAD *vs.* NHC | IgG | 0.334293 | 13.79% | 95.83% |
| JHU01178.P013G03 | FRS3 | Loess | Early-LUAD *vs.* NHC | IgG | 0.333971 | 12.07% | 95.83% |
| JHU19634.P182E05 | FOXH1 | Loess | Early-LUAD *vs.* NHC | IgG | 0.332794 | 18.97% | 100.00% |
| JHU19236.P195F01 | SETD8 | Loess | Early-LUAD *vs.* NHC | IgG | 0.330494 | 20.69% | 100.00% |
| JHU18615.P214C01 | DEK | Loess | Early-LUAD *vs.* NHC | IgG | 0.327098 | 20.69% | 95.83% |
| JHU06458.P068F06 | GEMIN8 | Loess | Early-LUAD *vs.* NHC | IgG | 0.324327 | 10.34% | 100.00% |
| JHU11344.P119E12 | DNAJC19 | Loess | Early-LUAD *vs.* NHC | IgG | 0.318694 | 20.69% | 100.00% |
| JHU12673.P234B06 | KCTD5 | Loess | Early-LUAD *vs.* NHC | IgG | 0.318387 | 15.52% | 100.00% |
| JHU15753.P165E12 | SLC36A3 | Loess | Early-LUAD *vs.* NHC | IgG | 0.31193 | 13.79% | 100.00% |
| JHU09863.P103C06 | RYBP | Loess | Early-LUAD *vs.* NHC | IgG | 0.311477 | 15.52% | 100.00% |
| JHU10245.P107C02 | RNF180 | Loess | Early-LUAD *vs.* NHC | IgG | 0.30807 | 24.14% | 100.00% |
| JHU06768.P071C10 | KJ900968*vs.*frag | Loess | Early-LUAD *vs.* NHC | IgG | 0.303422 | 17.24% | 100.00% |
| JHU07347.P080D02 | MOB1B | Loess | Early-LUAD *vs.* NHC | IgG | 0.302753 | 12.07% | 100.00% |
| JHU12947.P136E12 | FAM89B | Loess | Early-LUAD *vs.* NHC | IgG | 0.301829 | 12.07% | 100.00% |
| JHU18448.P212D10 | DAZL | Loess | Early-LUAD *vs.* NHC | IgG | 0.301348 | 15.52% | 95.83% |
| JHU15505.P163H09 | DIAPH3 | Loess | Early-LUAD *vs.* NHC | IgG | 0.300159 | 18.97% | 95.83% |
| JHU10661.P112H02 | PIFO | Loess | Early-LUAD *vs.* NHC | IgG | 0.299794 | 10.34% | 100.00% |
| JHU10020.P105G09 | ZGRF1 | Loess | Early-LUAD *vs.* NHC | IgG | 0.299327 | 17.24% | 95.83% |
| JHU12053.P188C11 | ZNF274 | Loess | Early-LUAD *vs.* NHC | IgG | 0.29909 | 17.24% | 100.00% |
| JHU24734.P242G07 | WHSC1 | Loess | Early-LUAD *vs.* NHC | IgG | 0.299062 | 10.34% | 100.00% |
| JHU07531.P079E06 | KIAA0391 | Loess | Early-LUAD *vs.* NHC | IgG | 0.298413 | 10.34% | 100.00% |
| JHU11894.P124D06 | ZNF75A | Loess | Early-LUAD *vs.* NHC | IgG | 0.289353 | 20.69% | 100.00% |
| JHU13040.P188D07 | CXXC1 | Loess | Early-LUAD *vs.* NHC | IgG | 0.289311 | 12.07% | 95.83% |
| JHU18602.P213H09 | TUB | Loess | Early-LUAD *vs.* NHC | IgG | 0.288978 | 13.79% | 95.83% |
| JHU19701.P183F06 | Q3zb72 | Loess | Early-LUAD *vs.* NHC | IgG | 0.285893 | 15.52% | 100.00% |
| JHU04472.P047D11 | RAB4A | Loess | Early-LUAD *vs.* NHC | IgG | 0.28551 | 10.34% | 95.83% |
| JHU06546.P069E06 | ERCC1 | Loess | Early-LUAD *vs.* NHC | IgG | 0.285098 | 24.14% | 100.00% |
| JHU06512.P068E02 | TEAD2 | Loess | Early-LUAD *vs.* NHC | IgG | 0.281406 | 13.79% | 100.00% |
| JHU18885.P226G01 | COQ10A | Loess | Early-LUAD *vs.* NHC | IgG | 0.274268 | 29.31% | 95.83% |
| JHU06632.P070D03 | C9orf85 | Loess | Early-LUAD *vs.* NHC | IgG | 0.273516 | 13.79% | 95.83% |
| JHU16868.P199F02 | IFNL3 | Loess | Early-LUAD *vs.* NHC | IgG | 0.272626 | 15.52% | 100.00% |
| JHU25029.P240G05 | ZNF550 | Loess | Early-LUAD *vs.* NHC | IgG | 0.272467 | 20.69% | 100.00% |
| JHU19234.P195H01 | RSPO4 | Loess | Early-LUAD *vs.* NHC | IgG | 0.267273 | 13.79% | 100.00% |
| JHU00177.P192G05 | RIOK3 | Loess | Early-LUAD *vs.* NHC | IgG | 0.267089 | 12.07% | 95.83% |
| JHU16339.P171F07 | WARS | Loess | Early-LUAD *vs.* NHC | IgG | 0.265086 | 13.79% | 95.83% |
| JHU01817.P186C05 | G3XAM8 | Loess | Early-LUAD *vs.* NHC | IgG | 0.258658 | 18.97% | 100.00% |
| JHU06598.P069E10 | PLAGL2 | Loess | Early-LUAD *vs.* NHC | IgG | 0.257794 | 12.07% | 100.00% |
| JHU10736.P112E05 | ZNF396 | Loess | Early-LUAD *vs.* NHC | IgG | 0.256912 | 13.79% | 95.83% |
| JHU19171.P195A10 | CCDC85A | Loess | Early-LUAD *vs.* NHC | IgG | 0.255718 | 20.69% | 100.00% |
| JHU14759.P155C07 | LBX2 | Loess | Early-LUAD *vs.* NHC | IgG | 0.254866 | 10.34% | 100.00% |
| JHU00798.P009G09 | DHX40 | Loess | Early-LUAD *vs.* NHC | IgG | 0.254422 | 12.07% | 100.00% |
| JHU07622.P178G05 | IQCA1 | Loess | Early-LUAD *vs.* NHC | IgG | 0.254164 | 29.31% | 91.67% |
| JHU10306.P108E12 | HRG | Loess | Early-LUAD *vs.* NHC | IgG | 0.2511 | 17.24% | 95.83% |
| JHU08503.P089B09 | PLCG2 | Loess | Early-LUAD *vs.* NHC | IgG | 0.246068 | 10.34% | 100.00% |
| JHU04406.P046H10 | TMEM45B | Loess | Early-LUAD *vs.* NHC | IgG | 0.245218 | 10.34% | 100.00% |
| JHU06990.P073F01 | SMARCAL1 | Loess | Early-LUAD *vs.* NHC | IgG | 0.241065 | 13.79% | 100.00% |
| JHU07312.P187E06 | CCBE1 | Loess | Early-LUAD *vs.* NHC | IgG | 0.238754 | 13.79% | 100.00% |
| JHU10433.P109E08 | PRR14 | Loess | Early-LUAD *vs.* NHC | IgG | 0.237806 | 12.07% | 100.00% |
| JHU12248.P128F10 | WBSCR22 | Loess | Early-LUAD *vs.* NHC | IgG | 0.237511 | 18.97% | 100.00% |
| JHU08206.P086C12 | LNX1 | Loess | Early-LUAD *vs.* NHC | IgG | 0.236511 | 18.97% | 95.83% |
| JHU05509.P058F02 | INTU | Loess | Early-LUAD *vs.* NHC | IgG | 0.235529 | 12.07% | 100.00% |
| JHU05997.P063E04 | HRASLS | Loess | Early-LUAD *vs.* NHC | IgG | 0.23145 | 10.34% | 100.00% |
| JHU10466.P110F04 | ASCC3 | Loess | Early-LUAD *vs.* NHC | IgG | 0.231407 | 13.79% | 100.00% |
| JHU06161.P065C06 | CBX8 | Loess | Early-LUAD *vs.* NHC | IgG | 0.230847 | 12.07% | 95.83% |
| JHU06606.P069B05 | TRMT10B | Loess | Early-LUAD *vs.* NHC | IgG | 0.230617 | 12.07% | 95.83% |
| JHU03229.P189G10 | MRPL30 | Loess | Early-LUAD *vs.* NHC | IgG | 0.22334 | 20.69% | 100.00% |
| JHU10756.P113H05 | BCORP1 | Loess | Early-LUAD *vs.* NHC | IgG | 0.213978 | 12.07% | 100.00% |
| JHU14971.P157C06 | SNRNP70 | Loess | Early-LUAD *vs.* NHC | IgG | 0.203741 | 24.14% | 95.83% |
| JHU07110.P075D10 | ANKRD13A | Loess | Early-LUAD *vs.* NHC | IgG | 0.200272 | 10.34% | 95.83% |
| JHU04876.P236G07 | SNAPIN | Loess | Early-LUAD *vs.* NHC | IgG | 0.19451 | 17.24% | 100.00% |
| JHU23967.P239G07 | ARHGAP9 | Loess | Early-LUAD *vs.* NHC | IgG | 0.186193 | 10.34% | 95.83% |
| JHU00425.P005D05 | H1F0 | Loess | Early-LUAD *vs.* Control | IgG | 0.585482 | 15.52% | 96.30% |
| JHU04415.P046E10 | ZNF397 | Loess | Early-LUAD *vs.* Control | IgG | 0.555512 | 13.79% | 92.59% |
| JHU04956.P052C05 | NFYC | Loess | Early-LUAD *vs.* Control | IgG | 0.505382 | 12.07% | 94.44% |
| JHU09419.P099G10 | NOL4L | Loess | Early-LUAD *vs.* Control | IgG | 0.463491 | 13.79% | 94.44% |
| JHU29369.P255C11 | FUOM | Loess | Early-LUAD *vs.* Control | IgG | 0.451657 | 20.69% | 90.74% |
| JHU10138.P106A10 | OMP | Loess | Early-LUAD *vs.* Control | IgG | 0.400559 | 10.34% | 96.30% |
| JHU11057.P116H11 | CCS | Loess | Early-LUAD *vs.* Control | IgG | 0.399047 | 13.79% | 94.44% |
| JHU04326.P046F06 | BIN1 | Loess | Early-LUAD *vs.* Control | IgG | 0.39812 | 15.52% | 98.15% |
| JHU11865.P124F11 | POLB | Loess | Early-LUAD *vs.* Control | IgG | 0.389052 | 17.24% | 94.44% |
| JHU00992.P231H05 | FABP5 | Loess | Early-LUAD *vs.* Control | IgG | 0.388834 | 13.79% | 96.30% |
| JHU27995.P247B03 | FBXL17 | Loess | Early-LUAD *vs.* Control | IgG | 0.388006 | 10.34% | 96.30% |
| JHU00131.P002E07 | JHU00131 | Loess | Early-LUAD *vs.* Control | IgG | 0.378016 | 12.07% | 98.15% |
| JHU09157.P096B06 | GPR108 | Loess | Early-LUAD *vs.* Control | IgG | 0.372841 | 12.07% | 94.44% |
| JHU21758.P240B04 | SYTL1 | Loess | Early-LUAD *vs.* Control | IgG | 0.364223 | 12.07% | 94.44% |
| JHU03189.P034D02 | COPS7A | Loess | Early-LUAD *vs.* Control | IgG | 0.333057 | 12.07% | 90.74% |
| JHU08471.P089D04 | FGF13 | Loess | Early-LUAD *vs.* Control | IgG | 0.305601 | 18.97% | 90.74% |
| JHU06900.P072C07 | TAAR2 | Loess | Early-LUAD *vs.* Control | IgG | 0.304816 | 10.34% | 100.00% |
| JHU11847.P124B04 | MAPRE1 | Loess | Early-LUAD *vs.* Control | IgG | 0.30281 | 13.79% | 94.44% |
| JHU15701.P253D07 | ADGRF1 | Loess | Early-LUAD *vs.* Control | IgG | 0.298057 | 12.07% | 96.30% |
| JHU19433.P197D06 | SGK223 | Loess | Early-LUAD *vs.* Control | IgG | 0.29357 | 18.97% | 94.44% |
| JHU14411.P151G03 | SPANXN4 | Loess | Early-LUAD *vs.* Control | IgG | 0.278553 | 15.52% | 94.44% |
| JHU01739.P189C07 | BTF3L4 | Loess | Early-LUAD *vs.* Control | IgG | 0.277336 | 10.34% | 100.00% |
| JHU25146.P240H01 | ALDH1L1 | Loess | Early-LUAD *vs.* Control | IgG | 0.266604 | 12.07% | 100.00% |
| JHU07702.P228A04 | EIF1B | Loess | Early-LUAD *vs.* Control | IgG | 0.265996 | 15.52% | 96.30% |
| JHU01921.P186D08 | 5-Sep | Loess | Early-LUAD *vs.* Control | IgG | 0.254783 | 15.52% | 96.30% |
| JHU06515.P068C03 | TNFRSF1B | Loess | Early-LUAD *vs.* Control | IgG | 0.254611 | 13.79% | 98.15% |
| JHU24641.P253B01 | BICC1 | Loess | Early-LUAD *vs.* Control | IgG | 0.253541 | 15.52% | 98.15% |
| JHU10692.P229D11 | KIAA1257 | Loess | Early-LUAD *vs.* Control | IgG | 0.251673 | 10.34% | 100.00% |
| JHU25609.P243D06 | VWF | Loess | Early-LUAD *vs.* Control | IgG | 0.2486 | 12.07% | 96.30% |
| JHU21937.P252B04 | PHF10 | Loess | Early-LUAD *vs.* Control | IgG | 0.246438 | 13.79% | 100.00% |
| JHU13551.P142E02 | PHF10 | Loess | Early-LUAD *vs.* Control | IgG | 0.238862 | 15.52% | 96.30% |
| JHU00596.P235G04 | CSRP2 | Loess | Early-LUAD *vs.* Control | IgG | 0.236351 | 12.07% | 94.44% |
| JHU01635.P227E01 | AFP | Loess | Early-LUAD *vs.* Control | IgG | 0.225433 | 15.52% | 96.30% |
| JHU12226.P128A01 | RDH14 | Loess | Early-LUAD *vs.* Control | IgG | 0.220772 | 17.24% | 98.15% |
| JHU07885.P083B06 | FAM206A | Loess | Early-LUAD *vs.* Control | IgG | 0.217648 | 15.52% | 96.30% |
| JHU06809.P071D06 | ZNF613 | Loess | Early-LUAD *vs.* Control | IgG | 0.214239 | 13.79% | 96.30% |
| JHU00862.P009H09 | ZNF302 | Loess | Early-LUAD *vs.* Control | IgG | 0.208344 | 10.34% | 92.59% |
| JHU08373.P228H09 | CEBPZ | Loess | Early-LUAD *vs.* Control | IgG | 0.198126 | 13.79% | 96.30% |
| JHU11577.P121C05 | RALGPS2 | Loess | Early-LUAD *vs.* Control | IgG | 0.197494 | 10.34% | 96.30% |
| JHU05576.P187C09 | FAM198B | Loess | Early-LUAD *vs.* Control | IgG | 0.18175 | 10.34% | 96.30% |
| JHU00037.P192F09 | HSP90B1 | Loess | Early-LUAD *vs.* Control | IgG | 0.164746 | 10.34% | 98.15% |
| JHU25137.P251G09 | SULF2 | Loess | Early-LUAD *vs.* Control | IgG | 0.164149 | 13.79% | 92.59% |
| JHU08536.P089B07 | KJ902887*vs.*frag | Loess | Early-LUAD *vs.* Control | IgG | 0.156689 | 18.97% | 92.59% |
| JHU16162.P011F03 | KIZ | Loess | Early-LUAD *vs.* BLD | IgM | 0.608228 | 10.53% | 96.67% |
| JHU10243.P107F10 | PRLR | Loess | Early-LUAD *vs.* BLD | IgM | 0.533804 | 10.53% | 100.00% |
| JHU05322.P056F01 | ISCA2 | Loess | Early-LUAD *vs.* BLD | IgM | 0.525396 | 7.89% | 90.00% |
| JHU07729.P081E11 | NAA38 | Loess | Early-LUAD *vs.* BLD | IgM | 0.495111 | 13.16% | 100.00% |
| JHU00171.P002E09 | PTPN6 | Loess | Early-LUAD *vs.* BLD | IgM | 0.485722 | 7.89% | 96.67% |
| JHU01017.P011D03 | ECE2 | Loess | Early-LUAD *vs.* BLD | IgM | 0.448142 | 10.53% | 96.67% |
| JHU02196.P023B05 | TCEAL3 | Loess | Early-LUAD *vs.* BLD | IgM | 0.44258 | 13.16% | 100.00% |
| JHU04218.P044A09 | UBA6 | Loess | Early-LUAD *vs.* BLD | IgM | 0.442509 | 7.89% | 96.67% |
| JHU12396.P130E08 | KCNE5 | Loess | Early-LUAD *vs.* BLD | IgM | 0.440656 | 7.89% | 100.00% |
| JHU01720.P176E02 | TGOLN2 | Loess | Early-LUAD *vs.* BLD | IgM | 0.431959 | 7.89% | 93.33% |
| JHU01649.P018C01 | ALX1 | Loess | Early-LUAD *vs.* BLD | IgM | 0.431154 | 10.53% | 100.00% |
| JHU13673.P143E05 | SPG7 | Loess | Early-LUAD *vs.* BLD | IgM | 0.426932 | 10.53% | 93.33% |
| JHU10237.P107E06 | PCDHGB1 | Loess | Early-LUAD *vs.* BLD | IgM | 0.421565 | 13.16% | 96.67% |
| JHU09153.P096E05 | FXYD6 | Loess | Early-LUAD *vs.* BLD | IgM | 0.415011 | 10.53% | 100.00% |
| JHU02279.P024D09 | RPL36AL | Loess | Early-LUAD *vs.* BLD | IgM | 0.413831 | 10.53% | 96.67% |
| JHU01949.P021B11 | EIF1AY | Loess | Early-LUAD *vs.* BLD | IgM | 0.406523 | 10.53% | 100.00% |
| JHU08459.P089E05 | CDKN1B | Loess | Early-LUAD *vs.* BLD | IgM | 0.398931 | 10.53% | 100.00% |
| JHU01806.P019D05 | SMCP | Loess | Early-LUAD *vs.* BLD | IgM | 0.397562 | 7.89% | 96.67% |
| JHU07851.P082C09 | SUV39H2 | Loess | Early-LUAD *vs.* BLD | IgM | 0.39527 | 7.89% | 96.67% |
| JHU05306.P056E05 | DTD1 | Loess | Early-LUAD *vs.* BLD | IgM | 0.371158 | 7.89% | 86.67% |
| JHU06129.P064A08 | TBK1 | Loess | Early-LUAD *vs.* BLD | IgM | 0.370599 | 7.89% | 93.33% |
| JHU11139.P117F09 | BCL7B | Loess | Early-LUAD *vs.* BLD | IgM | 0.370011 | 18.42% | 100.00% |
| JHU00555.P006A11 | RPUSD4 | Loess | Early-LUAD *vs.* BLD | IgM | 0.369851 | 13.16% | 100.00% |
| JHU01629.P017D01 | UCHL5 | Loess | Early-LUAD *vs.* BLD | IgM | 0.361232 | 7.89% | 100.00% |
| JHU06930.P073D06 | CSF3R | Loess | Early-LUAD *vs.* BLD | IgM | 0.358476 | 7.89% | 96.67% |
| JHU06834.P072F06 | CCNE2 | Loess | Early-LUAD *vs.* BLD | IgM | 0.355909 | 7.89% | 100.00% |
| JHU07488.P079C03 | ABCC10*vs.*frag | Loess | Early-LUAD *vs.* BLD | IgM | 0.352491 | 10.53% | 96.67% |
| JHU05163.P054F09 | SPZ1 | Loess | Early-LUAD *vs.* BLD | IgM | 0.35133 | 10.53% | 96.67% |
| JHU13699.P144B05 | URI1 | Loess | Early-LUAD *vs.* BLD | IgM | 0.348933 | 7.89% | 96.67% |
| JHU19419.P197F09 | PPP1R3F | Loess | Early-LUAD *vs.* BLD | IgM | 0.348565 | 7.89% | 100.00% |
| JHU11633.P122F12 | DECR1 | Loess | Early-LUAD *vs.* BLD | IgM | 0.344927 | 7.89% | 96.67% |
| JHU08654.P091E03 | CCNF | Loess | Early-LUAD *vs.* BLD | IgM | 0.339912 | 7.89% | 96.67% |
| JHU06636.P070A01 | DDR1 | Loess | Early-LUAD *vs.* BLD | IgM | 0.337842 | 7.89% | 100.00% |
| JHU05456.P057D08 | STRAP | Loess | Early-LUAD *vs.* BLD | IgM | 0.336387 | 7.89% | 100.00% |
| JHU01837.P020F05 | TPGS2 | Loess | Early-LUAD *vs.* BLD | IgM | 0.330169 | 7.89% | 96.67% |
| JHU04517.P048E12 | ARPC5L | Loess | Early-LUAD *vs.* BLD | IgM | 0.327807 | 7.89% | 96.67% |
| JHU02649.P028F01 | OS9 | Loess | Early-LUAD *vs.* BLD | IgM | 0.323826 | 7.89% | 96.67% |
| JHU06018.P063C03 | OOSP2 | Loess | Early-LUAD *vs.* BLD | IgM | 0.320948 | 7.89% | 93.33% |
| JHU07797.P082C12 | COL4A6 | Loess | Early-LUAD *vs.* BLD | IgM | 0.319499 | 10.53% | 100.00% |
| JHU05396.P057F08 | DYNC2LI1 | Loess | Early-LUAD *vs.* BLD | IgM | 0.317849 | 7.89% | 93.33% |
| JHU00789.P009D10 | CD99L2 | Loess | Early-LUAD *vs.* BLD | IgM | 0.311801 | 7.89% | 93.33% |
| JHU06950.P073D05 | JUP | Loess | Early-LUAD *vs.* BLD | IgM | 0.311662 | 18.42% | 96.67% |
| JHU10811.P113D07 | SFTA2 | Loess | Early-LUAD *vs.* BLD | IgM | 0.308434 | 7.89% | 100.00% |
| JHU08576.P096G10 | EPB41L1 | Loess | Early-LUAD *vs.* BLD | IgM | 0.303598 | 7.89% | 100.00% |
| JHU10472.P110E09 | BNIPL | Loess | Early-LUAD *vs.* BLD | IgM | 0.298948 | 7.89% | 93.33% |
| JHU05478.P058E06 | ANKMY1 | Loess | Early-LUAD *vs.* BLD | IgM | 0.298423 | 13.16% | 96.67% |
| JHU08480.P089C02 | HOXC5 | Loess | Early-LUAD *vs.* BLD | IgM | 0.2953 | 7.89% | 96.67% |
| JHU06715.P070C02 | ZNF695 | Loess | Early-LUAD *vs.* BLD | IgM | 0.286832 | 7.89% | 100.00% |
| JHU05605.P059D07 | GNAZ | Loess | Early-LUAD *vs.* BLD | IgM | 0.285775 | 10.53% | 93.33% |
| JHU06038.P063D02 | TRAPPC6B | Loess | Early-LUAD *vs.* BLD | IgM | 0.28266 | 7.89% | 96.67% |
| JHU07297.P080F06 | ADH6 | Loess | Early-LUAD *vs.* BLD | IgM | 0.282592 | 7.89% | 100.00% |
| JHU12444.P131E08 | AY312367*vs.*frag | Loess | Early-LUAD *vs.* BLD | IgM | 0.281044 | 7.89% | 96.67% |
| JHU13589.P142F08 | ZNF446 | Loess | Early-LUAD *vs.* BLD | IgM | 0.278403 | 10.53% | 93.33% |
| JHU08532.P089E06 | UBOX5 | Loess | Early-LUAD *vs.* BLD | IgM | 0.277937 | 7.89% | 100.00% |
| JHU05790.P061D12 | MPHOSPH8*vs.*frag | Loess | Early-LUAD *vs.* BLD | IgM | 0.268781 | 7.89% | 90.00% |
| JHU06899.P072E01 | EPPIN | Loess | Early-LUAD *vs.* BLD | IgM | 0.264185 | 10.53% | 96.67% |
| JHU10400.P109F08 | KCNA6 | Loess | Early-LUAD *vs.* BLD | IgM | 0.258025 | 7.89% | 93.33% |
| JHU06663.P070D07 | NCOA4 | Loess | Early-LUAD *vs.* BLD | IgM | 0.25787 | 7.89% | 100.00% |
| JHU08155.P085D06 | DAW1 | Loess | Early-LUAD *vs.* BLD | IgM | 0.254436 | 10.53% | 100.00% |
| JHU12392.P130G08 | IL17D | Loess | Early-LUAD *vs.* BLD | IgM | 0.251024 | 7.89% | 100.00% |
| JHU13268.P139F10 | OR2C3 | Loess | Early-LUAD *vs.* BLD | IgM | 0.246946 | 13.16% | 96.67% |
| JHU03410.P036E01 | LOC401152*vs.*frag | Loess | Early-LUAD *vs.* BLD | IgM | 0.241457 | 7.89% | 100.00% |
| JHU02831.P030D06 | LYRM4 | Loess | Early-LUAD *vs.* BLD | IgM | 0.240593 | 7.89% | 96.67% |
| JHU06835.P072D02 | CDA | Loess | Early-LUAD *vs.* BLD | IgM | 0.233827 | 7.89% | 90.00% |
| JHU06901.P072E09 | TBC1D16 | Loess | Early-LUAD *vs.* BLD | IgM | 0.223469 | 15.79% | 90.00% |
| JHU06367.P067C11 | TTC23L | Loess | Early-LUAD *vs.* BLD | IgM | 0.221118 | 18.42% | 96.67% |
| JHU06576.P069C05 | OR2F1 | Loess | Early-LUAD *vs.* BLD | IgM | 0.209684 | 7.89% | 96.67% |
| JHU11949.P125B11 | LRRC55 | Loess | Early-LUAD *vs.* BLD | IgM | 0.20378 | 7.89% | 96.67% |
| JHU09444.P099F02 | HDHD3 | Loess | Early-LUAD *vs.* BLD | IgM | 0.184529 | 13.16% | 95.83% |
| JHU02685.P028A08 | DCTPP1 | Supplement methods | Early-LUAD *vs.* NHC | IgM | 2.20474 | 15.79% | 100.00% |
| JHU06755.P071C01 | GDA | Supplement methods | Early-LUAD *vs.* NHC | IgM | 2.923049 | 13.16% | 100.00% |
| JHU00319.P004A10 | HN1 | Loess | Early-LUAD *vs.* NHC | IgM | 0.579859 | 18.42% | 100.00% |
| JHU09728.P102A03 | HBD | Loess | Early-LUAD *vs.* NHC | IgM | 0.521504 | 7.89% | 100.00% |
| JHU19230.P195E04 | QSOX1 | Loess | Early-LUAD *vs.* NHC | IgM | 0.506613 | 10.53% | 95.83% |
| JHU06128.P064B10 | TAGLN2 | Loess | Early-LUAD *vs.* NHC | IgM | 0.503812 | 10.53% | 100.00% |
| JHU01889.P020A06 | PLEK | Loess | Early-LUAD *vs.* NHC | IgM | 0.492308 | 36.84% | 95.83% |
| JHU04533.P048E11 | CARHSP1 | Loess | Early-LUAD *vs.* NHC | IgM | 0.488188 | 13.16% | 100.00% |
| JHU16162.P011H03 | KIZ | Loess | Early-LUAD *vs.* NHC | IgM | 0.474844 | 7.89% | 100.00% |
| JHU14683.P154C02 | PSD | Loess | Early-LUAD *vs.* NHC | IgM | 0.45998 | 18.42% | 100.00% |
| JHU13265.P139C07 | NUDT4 | Loess | Early-LUAD *vs.* NHC | IgM | 0.45871 | 7.89% | 95.83% |
| JHU00371.P004C11 | TAGLN2 | Loess | Early-LUAD *vs.* NHC | IgM | 0.457968 | 10.53% | 100.00% |
| JHU15907.P167B08 | IST1 | Loess | Early-LUAD *vs.* NHC | IgM | 0.455272 | 7.89% | 100.00% |
| JHU13860.P145A07 | RASSF1 | Loess | Early-LUAD *vs.* NHC | IgM | 0.440363 | 18.42% | 95.83% |
| JHU03846.P041A08 | ACBD7 | Loess | Early-LUAD *vs.* NHC | IgM | 0.438 | 13.16% | 95.83% |
| JHU00126.P002A10 | DUSP3 | Loess | Early-LUAD *vs.* NHC | IgM | 0.436567 | 18.42% | 95.83% |
| JHU03033.P032F12 | MGMT | Loess | Early-LUAD *vs.* NHC | IgM | 0.435182 | 7.89% | 95.83% |
| JHU00603.P007G10 | EMCN | Loess | Early-LUAD *vs.* NHC | IgM | 0.4318 | 15.79% | 100.00% |
| JHU11081.P116A03 | MED29 | Loess | Early-LUAD *vs.* NHC | IgM | 0.425523 | 13.16% | 100.00% |
| JHU00761.P008F12 | TRIM9 | Loess | Early-LUAD *vs.* NHC | IgM | 0.406379 | 15.79% | 100.00% |
| JHU14685.P154C06 | RELB | Loess | Early-LUAD *vs.* NHC | IgM | 0.400356 | 7.89% | 95.83% |
| JHU14580.P188F08 | PFN2 | Loess | Early-LUAD *vs.* NHC | IgM | 0.395259 | 13.16% | 100.00% |
| JHU08730.P178H10 | VPS13B*vs.*frag | Loess | Early-LUAD *vs.* NHC | IgM | 0.393511 | 10.53% | 100.00% |
| JHU00977.P011G01 | PCIF1 | Loess | Early-LUAD *vs.* NHC | IgM | 0.386107 | 7.89% | 100.00% |
| JHU06268.P066B10 | KCMF1 | Loess | Early-LUAD *vs.* NHC | IgM | 0.383682 | 7.89% | 100.00% |
| JHU14718.P155D07 | BNIP1 | Loess | Early-LUAD *vs.* NHC | IgM | 0.380412 | 18.42% | 100.00% |
| JHU04989.P052F08 | ZC3H7A | Loess | Early-LUAD *vs.* NHC | IgM | 0.378909 | 13.16% | 95.83% |
| JHU04463.P047C12 | PDLIM7 | Loess | Early-LUAD *vs.* NHC | IgM | 0.370103 | 10.53% | 100.00% |
| JHU15569.P163C01 | TMOD2 | Loess | Early-LUAD *vs.* NHC | IgM | 0.369179 | 15.79% | 95.83% |
| JHU19471.P198C12 | EIF2AK3 | Loess | Early-LUAD *vs.* NHC | IgM | 0.367893 | 7.89% | 95.83% |
| JHU00322.P004A07 | IFI35 | Loess | Early-LUAD *vs.* NHC | IgM | 0.365843 | 7.89% | 100.00% |
| JHU10033.P105B08 | PADI4 | Loess | Early-LUAD *vs.* NHC | IgM | 0.36534 | 18.42% | 100.00% |
| JHU03771.P040B10 | CDK2AP2 | Loess | Early-LUAD *vs.* NHC | IgM | 0.365147 | 13.16% | 95.83% |
| JHU00695.P008C11 | DAG1 | Loess | Early-LUAD *vs.* NHC | IgM | 0.35322 | 7.89% | 100.00% |
| JHU08758.P092D07 | COPZ2 | Loess | Early-LUAD *vs.* NHC | IgM | 0.351379 | 10.53% | 100.00% |
| JHU00197.P003A02 | ARF6 | Loess | Early-LUAD *vs.* NHC | IgM | 0.350512 | 13.16% | 100.00% |
| JHU00874.P010F12 | BAZ2B | Loess | Early-LUAD *vs.* NHC | IgM | 0.34694 | 7.89% | 100.00% |
| JHU00699.P008B12 | ELAC1 | Loess | Early-LUAD *vs.* NHC | IgM | 0.34411 | 15.79% | 100.00% |
| JHU02166.P023E04 | NFE2 | Loess | Early-LUAD *vs.* NHC | IgM | 0.342888 | 7.89% | 100.00% |
| JHU00752.P008D11 | TADA3 | Loess | Early-LUAD *vs.* NHC | IgM | 0.339712 | 10.53% | 100.00% |
| JHU14459.P152F10 | BC132980.1*vs.*frag | Loess | Early-LUAD *vs.* NHC | IgM | 0.339496 | 10.53% | 95.83% |
| JHU03210.P034A07 | GYG2 | Loess | Early-LUAD *vs.* NHC | IgM | 0.331192 | 7.89% | 100.00% |
| JHU01959.P021A09 | GMNN | Loess | Early-LUAD *vs.* NHC | IgM | 0.326031 | 13.16% | 100.00% |
| JHU01725.P018B12 | EZR | Loess | Early-LUAD *vs.* NHC | IgM | 0.323425 | 7.89% | 95.83% |
| JHU08390.P088D10 | GPR155 | Loess | Early-LUAD *vs.* NHC | IgM | 0.32018 | 10.53% | 95.83% |
| JHU01230.P013H08 | SUB1 | Loess | Early-LUAD *vs.* NHC | IgM | 0.317181 | 10.53% | 100.00% |
| JHU10583.P233D11 | CSK | Loess | Early-LUAD *vs.* NHC | IgM | 0.315439 | 7.89% | 95.83% |
| JHU10750.P112F12 | XR*vs.*922687.1*vs.*frag | Loess | Early-LUAD *vs.* NHC | IgM | 0.315307 | 7.89% | 100.00% |
| JHU16655.P206G03 | HOXD12 | Loess | Early-LUAD *vs.* NHC | IgM | 0.311609 | 7.89% | 100.00% |
| JHU02620.P028A07 | ECT2*vs.*frag | Loess | Early-LUAD *vs.* NHC | IgM | 0.308596 | 26.32% | 95.83% |
| JHU17564.P218D07 | OR8U8 | Loess | Early-LUAD *vs.* NHC | IgM | 0.295707 | 13.16% | 100.00% |
| JHU06778.P071C06 | NR1D1 | Loess | Early-LUAD *vs.* NHC | IgM | 0.294901 | 13.16% | 100.00% |
| JHU01275.P014G10 | CMSS1 | Loess | Early-LUAD *vs.* NHC | IgM | 0.291303 | 7.89% | 100.00% |
| JHU09933.P104G10 | NCBP2-AS2 | Loess | Early-LUAD *vs.* NHC | IgM | 0.290457 | 7.89% | 100.00% |
| JHU16385.P172B05 | EZR | Loess | Early-LUAD *vs.* NHC | IgM | 0.287863 | 7.89% | 100.00% |
| JHU17037.P201B10 | TMEM257 | Loess | Early-LUAD *vs.* NHC | IgM | 0.285264 | 13.16% | 100.00% |
| JHU08112.P085D11 | MSX2 | Loess | Early-LUAD *vs.* NHC | IgM | 0.28404 | 13.16% | 95.83% |
| JHU01878.P020D04 | LINC00312 | Loess | Early-LUAD *vs.* NHC | IgM | 0.281463 | 10.53% | 100.00% |
| JHU03413.P036B01 | LYPLAL1 | Loess | Early-LUAD *vs.* NHC | IgM | 0.280121 | 10.53% | 100.00% |
| JHU00157.P002G11 | PPP1R14A | Loess | Early-LUAD *vs.* NHC | IgM | 0.278247 | 15.79% | 100.00% |
| JHU02920.P031A07 | HOXA5 | Loess | Early-LUAD *vs.* NHC | IgM | 0.274691 | 7.89% | 100.00% |
| JHU06500.P068A07 | REEP6 | Loess | Early-LUAD *vs.* NHC | IgM | 0.260037 | 10.53% | 100.00% |
| JHU00823.P009G03 | N6AMT1 | Loess | Early-LUAD *vs.* NHC | IgM | 0.256265 | 7.89% | 100.00% |
| JHU07284.P253B07 | STK16 | Loess | Early-LUAD *vs.* NHC | IgM | 0.25624 | 7.89% | 100.00% |
| JHU00049.P001G07 | MBIP | Loess | Early-LUAD *vs.* NHC | IgM | 0.253132 | 13.16% | 100.00% |
| JHU00461.P005B01 | SLC1A7 | Loess | Early-LUAD *vs.* NHC | IgM | 0.251107 | 10.53% | 100.00% |
| JHU01764.P019D07 | FAM92A1 | Loess | Early-LUAD *vs.* NHC | IgM | 0.25017 | 7.89% | 95.83% |
| JHU01203.P013B07 | PIGT | Loess | Early-LUAD *vs.* NHC | IgM | 0.247007 | 7.89% | 100.00% |
| JHU06097.P064C06 | KIF2A | Loess | Early-LUAD *vs.* NHC | IgM | 0.246182 | 15.79% | 100.00% |
| JHU00771.P009H01 | ANTXR1 | Loess | Early-LUAD *vs.* NHC | IgM | 0.239531 | 7.89% | 95.83% |
| JHU12310.P129A10 | MYBBP1A | Loess | Early-LUAD *vs.* NHC | IgM | 0.229387 | 13.16% | 100.00% |
| JHU15285.P160F08 | WDR34 | Loess | Early-LUAD *vs.* NHC | IgM | 0.218189 | 10.53% | 100.00% |
| JHU16004.P001C10 | KPNA1 | Loess | Early-LUAD *vs.* Control | IgM | 0.374258 | 7.89% | 98.15% |
| JHU00521.P006C12 | GCHFR | Loess | Early-LUAD *vs.* Control | IgM | 0.373277 | 15.79% | 94.44% |
| JHU02445.P026A05 | IFT20 | Loess | Early-LUAD *vs.* Control | IgM | 0.366982 | 7.89% | 98.15% |
| JHU01478.P016D08 | FLOT2 | Loess | Early-LUAD *vs.* Control | IgM | 0.346182 | 13.16% | 96.30% |
| JHU00941.P010C02 | SC5D | Loess | Early-LUAD *vs.* Control | IgM | 0.338596 | 13.16% | 94.44% |
| JHU00728.P008C03 | HPGDS | Loess | Early-LUAD *vs.* Control | IgM | 0.3174 | 13.16% | 98.15% |
| JHU01956.P021G08 | FKBP14 | Loess | Early-LUAD *vs.* Control | IgM | 0.310185 | 15.79% | 96.30% |
| JHU01501.P016C01 | RPRD1A | Loess | Early-LUAD *vs.* Control | IgM | 0.302746 | 10.53% | 94.44% |
| JHU02601.P028B10 | CD2BP2 | Loess | Early-LUAD *vs.* Control | IgM | 0.294825 | 10.53% | 98.15% |
| JHU06344.P067E06 | C1orf210 | Loess | Early-LUAD *vs.* Control | IgM | 0.290314 | 7.89% | 96.30% |
| JHU00769.P009E02 | 1-Sep | Loess | Early-LUAD *vs.* Control | IgM | 0.289073 | 7.89% | 96.30% |
| JHU15603.P164B03 | GBP5 | Loess | Early-LUAD *vs.* Control | IgM | 0.286608 | 7.89% | 98.15% |
| JHU02151.P023H04 | HMGN2*vs.*frag | Loess | Early-LUAD *vs.* Control | IgM | 0.259935 | 7.89% | 92.59% |
| JHU06644.P070B06 | H1FOO | Loess | Early-LUAD *vs.* Control | IgM | 0.249784 | 7.89% | 98.15% |
| JHU00169.P002A09 | PTPN11 | Loess | Early-LUAD *vs.* Control | IgM | 0.241887 | 7.89% | 94.44% |
| JHU05091.P054D02 | GPN2 | Loess | Early-LUAD *vs.* Control | IgM | 0.230392 | 10.53% | 94.44% |
| JHU15195.P160C09 | ASPA | Loess | Early-LUAD *vs.* Control | IgM | 0.221803 | 7.89% | 90.74% |
| JHU06862.P072C06 | IYD | Loess | Early-LUAD *vs.* Control | IgM | 0.217531 | 7.89% | 98.15% |
| JHU07113.P075A11 | BEST1 | Loess | Early-LUAD *vs.* Control | IgM | 0.198081 | 10.53% | 100.00% |
| JHU13157.P138C01 | KLK4 | Loess | Early-LUAD *vs.* Control | IgM | 0.172243 | 10.53% | 96.30% |
| JHU03539.P037B07 | TIMM17A | Loess | Early-LUAD *vs.* Control | IgM | 0.14031 | 10.53% | 96.30% |
| JHU18291.P210C01 | MAGEA1 | literature | - | reported | PMID 29308305 | | |
| JHU04491.P234C02 | SOX2 | literature | - | reported | PMID 29308305 | | |
| JHU04788.P050D05 | TP53 | literature | - | reported | PMID 29308305 | | |
| JHU05218.P055C11 | GAGE7 | literature | - | reported | PMID 29308305 | | |
| JHU04505.P047D12 | UCHL1/PGP9.5 | literature | - | reported | PMID 29308305 | | |
| JHU16271.P171C04 | CAGE/DDX53 | literature | - | reported | PMID 29308305 | | |
| JHU29290.P266D01 | GBU4-5 | literature | - | reported | PMID 29308305 | | |
| JHU17795.P221D02 | CTAG1A | literature | - | reported | PMID 29021294 | | |
| JHU05591.P059G09 | HuD/ELAVL4 | literature | - | reported | PMID 32732334 | | |
| JHU01014.P011G12 | MAGEA4 | literature | - | reported | PMID 32732334 | | |
| JHU08261.P087E10 | ANXA1 | literature | - | reported | PMID 21733826 | | |
| JHU13409.P141B07 | BCL7A | literature | - | reported | PMID 31924693 | | |
| JHU19543.P198C04 | TRIM33 | literature | - | reported | PMID 31924693 | | |
| JHU07505.P079H09 | DDX4 | literature | - | reported | PMID 31924693 | | |
| JHU06193.P065C05 | MAGEC2 | literature | - | reported | PMID 31924693 | | |
| JHU00417.P005D10 | ETHE1 | literature | - | reported | PMID 31924693 | | |
| JHU09539.P100C07 | GIP | literature | - | reported | PMID 31924693 | | |
| JHU02290.P227B03 | TEX264 | literature | - | reported | PMID 31924693 | | |
| JHU00792.P009C04 | CLDN2 | literature | - | reported | PMID 31924693 | | |
| JHU02227.P024H10 | NSG1 | literature | - | reported | PMID 31924693 | | |
| JHU26821.P245G03 | AQP4 | AAg Atlas database | - | reported | http://biokb.ncpsb.org.cn/aagatlas_portal/index.php | | |
| JHU12928.P238C10 | AZGP1 | AAg Atlas database | - | reported | http://biokb.ncpsb.org.cn/aagatlas_portal/index.php | | |
| JHU02029.P186E08 | CALR | AAg Atlas database | - | reported | http://biokb.ncpsb.org.cn/aagatlas_portal/index.php | | |
| JHU09494.P099G06 | UBQLN1 | AAg Atlas database | - | reported | http://biokb.ncpsb.org.cn/aagatlas_portal/index.php | | |
| JHU04891.P225G04 | XAGE1A | AAg Atlas database | - | reported | http://biokb.ncpsb.org.cn/aagatlas_portal/index.php | | |
| JHU18243.P210A09 | CAGE1 | AAg Atlas database | - | reported | http://biokb.ncpsb.org.cn/aagatlas_portal/index.php | | |
