# Supplementary figures and images for: A Novel IgG–IgM Autoantibody Panel Enhances Detection of Early-stage Lung Adenocarcinoma from Benign Nodules
Source: Genomics Proteomics Bioinformatics. 2024 Dec 11;22(6):qzae085. doi: 10.1093/gpbjnl/qzae085 (PMC12032526; doi:10.1093/gpbjnl/qzae085)

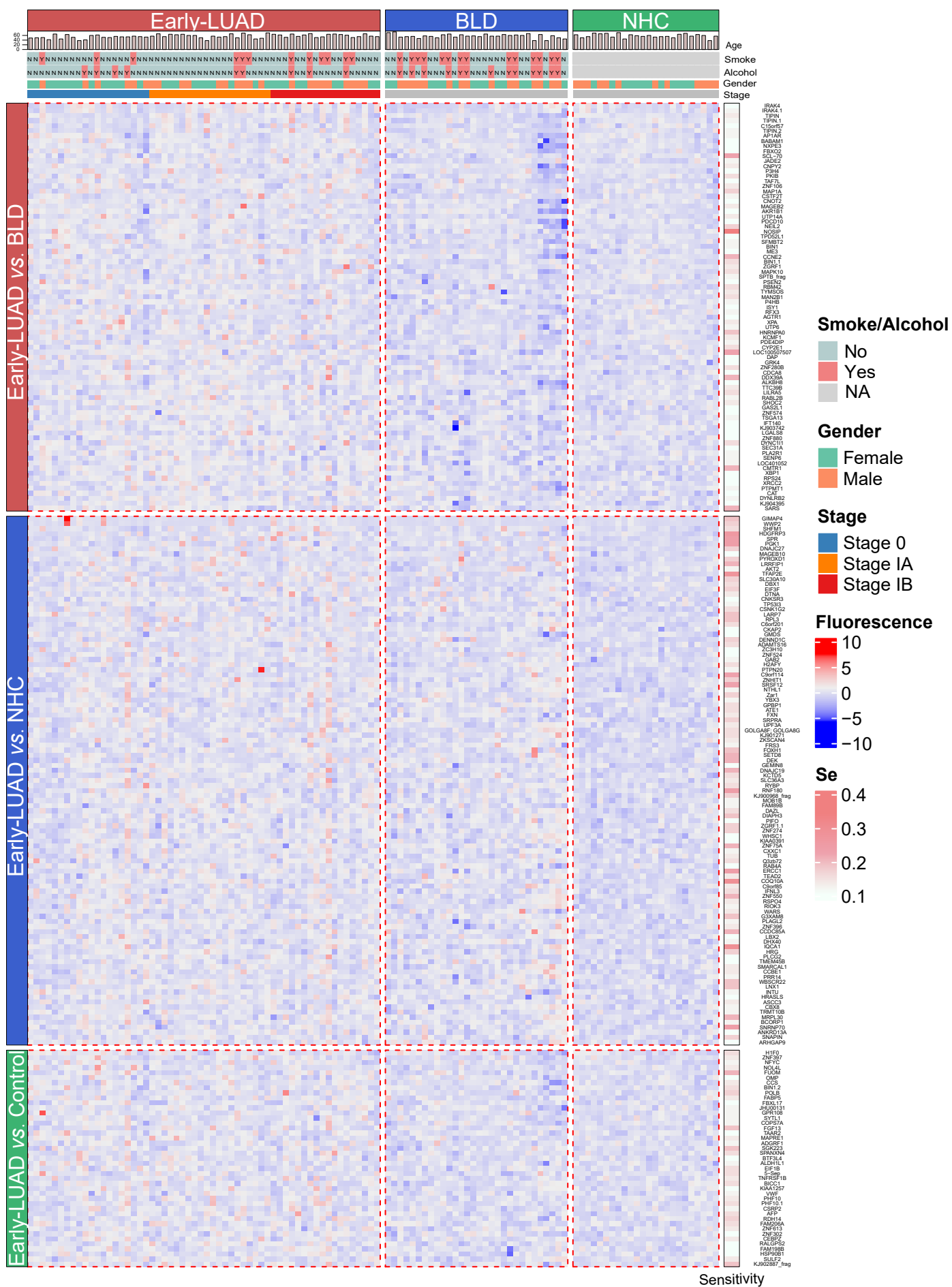

Supplement: qzae085_Supplementary_Data [file qzae085_supplementary_data.zip › FigureS2.pdf]

A

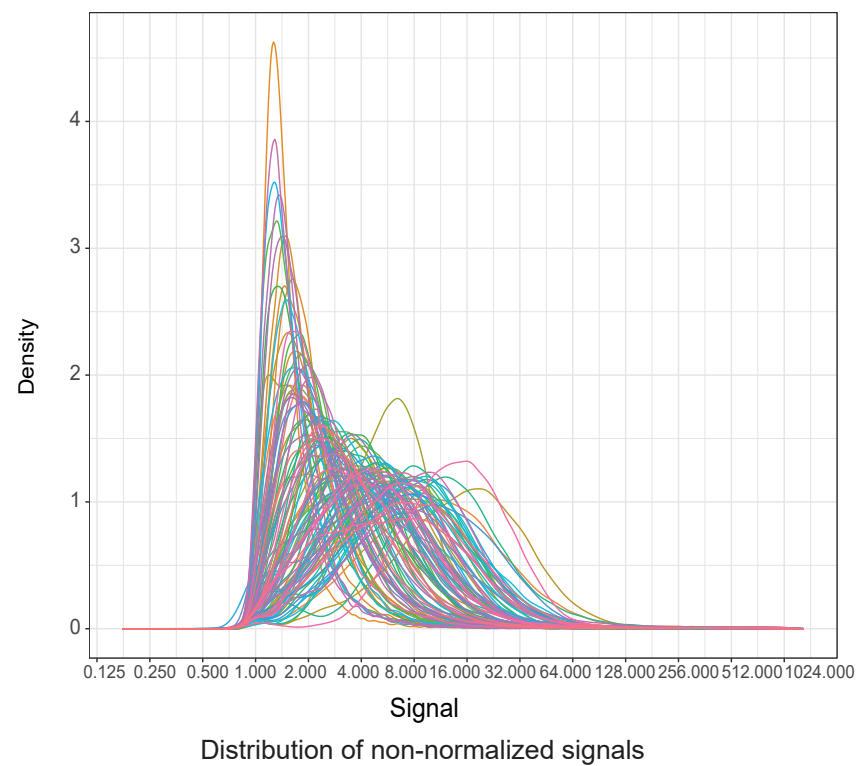

B

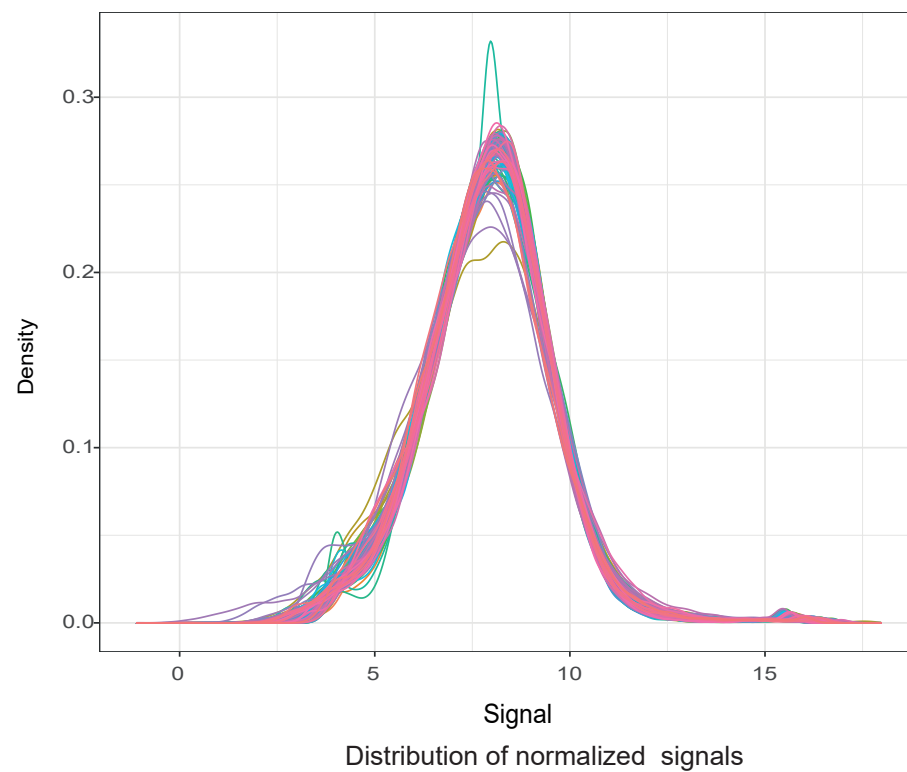

C

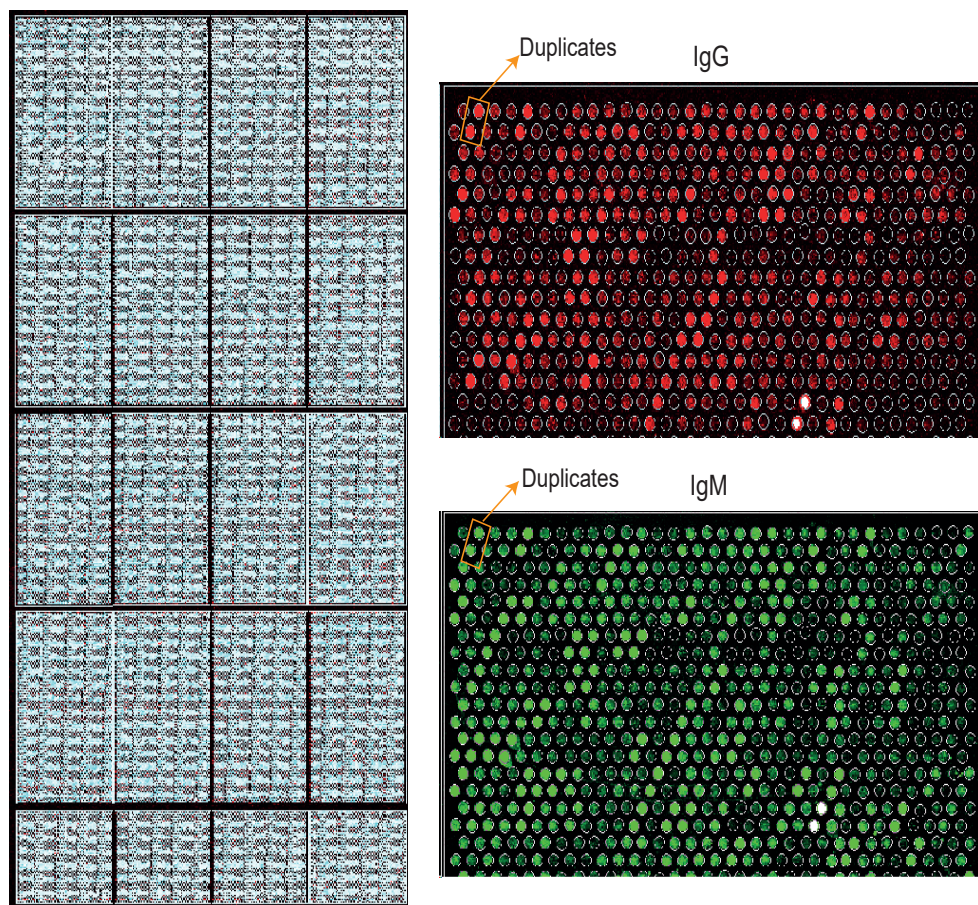

D

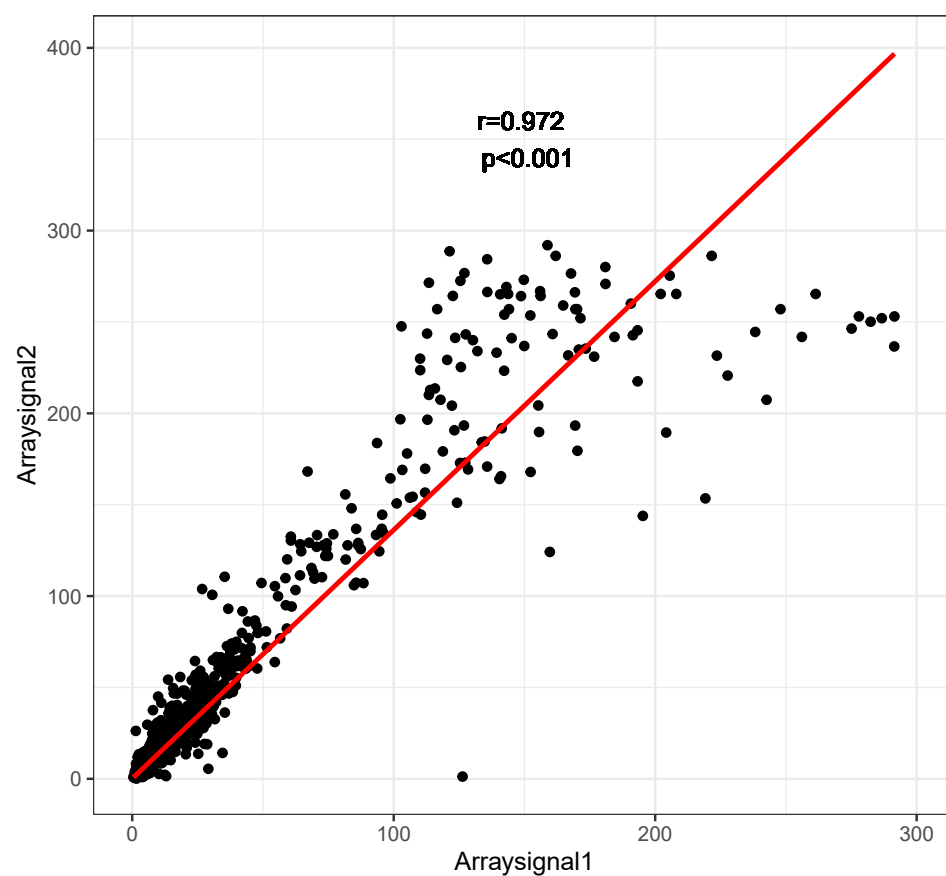

Supplement: qzae085_Supplementary_Data [file qzae085_supplementary_data.zip › FigureS1.pdf]

# A

## GO enrichment

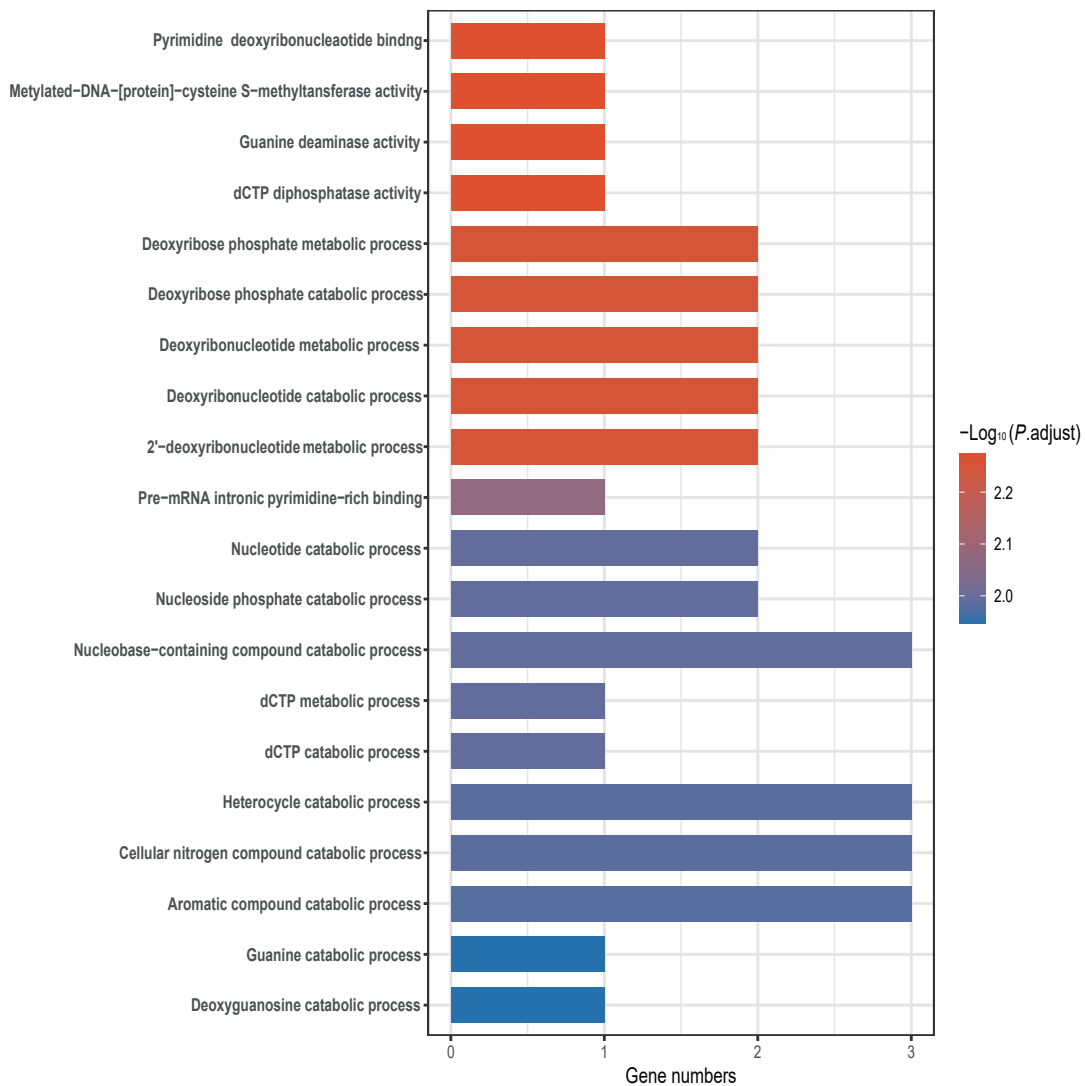

# B

## KEGG pathways enrichment

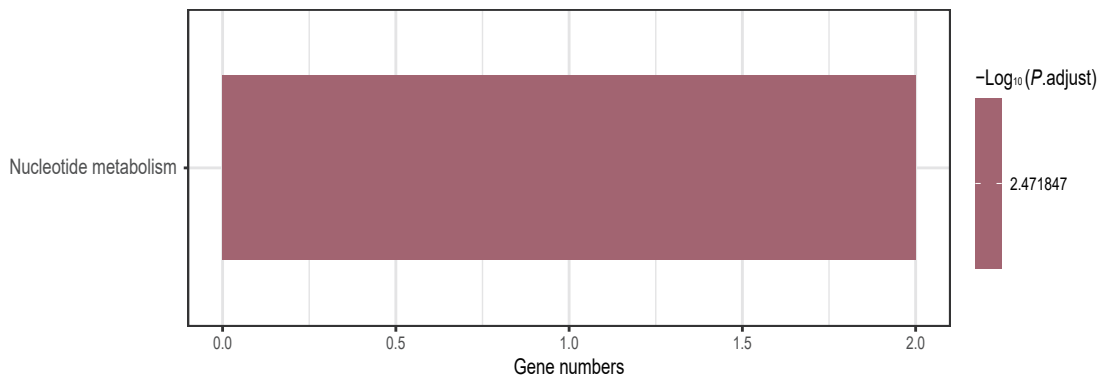

Supplement: qzae085_Supplementary_Data [file qzae085_supplementary_data.zip › FigureS6.pdf]

A

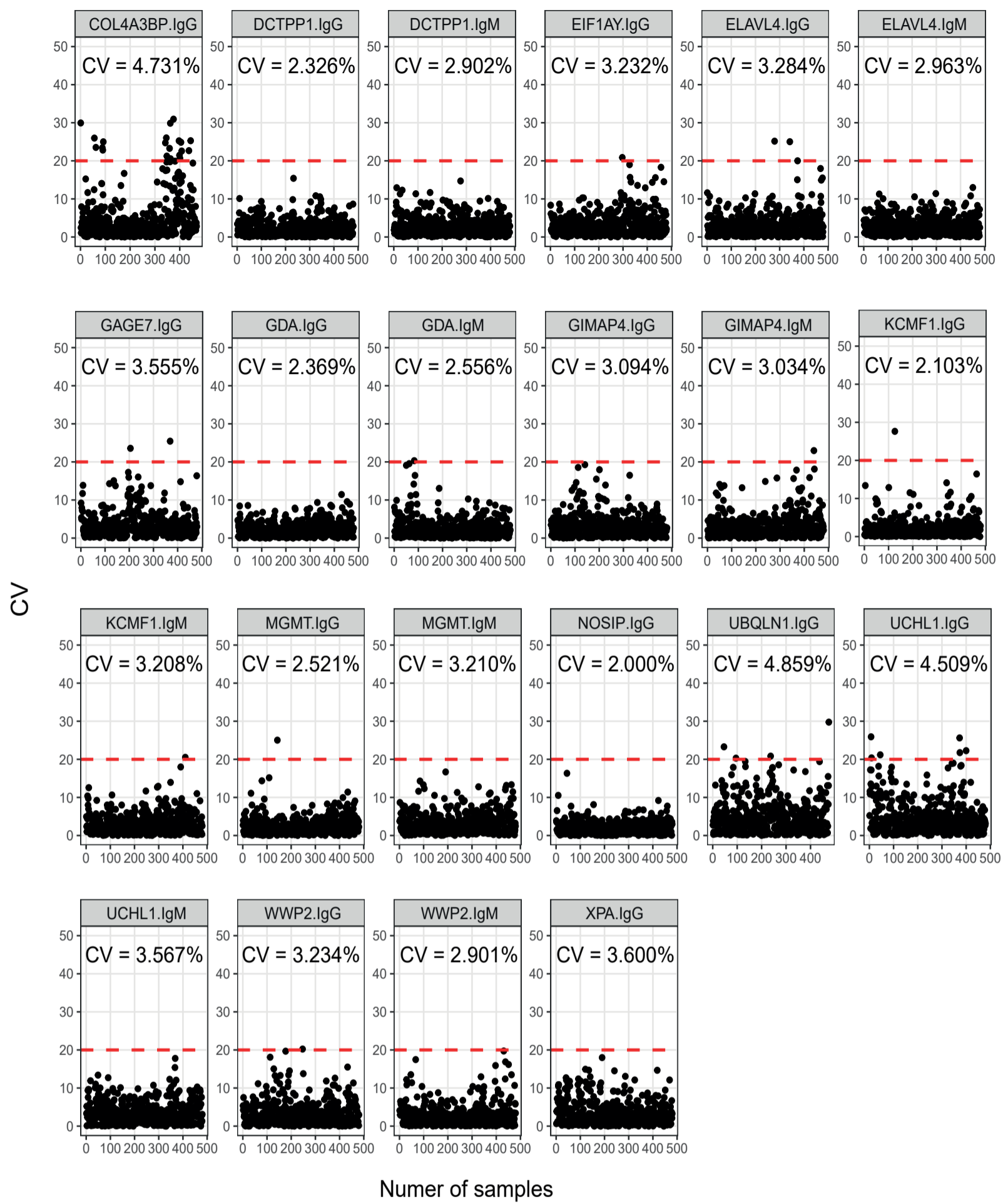

B

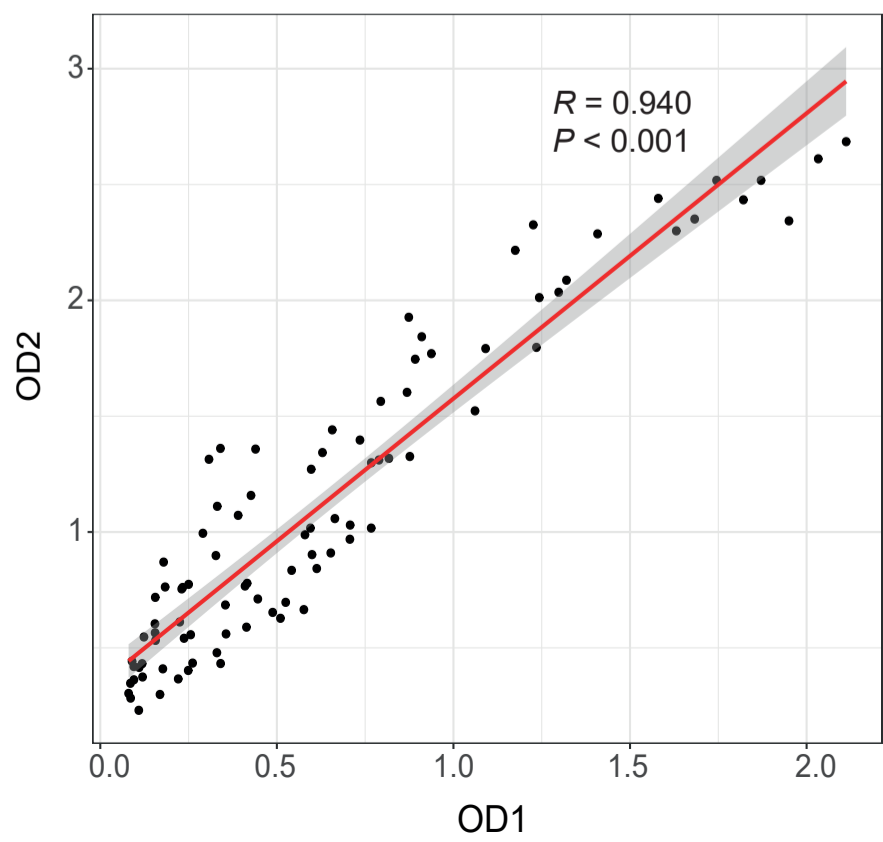

C

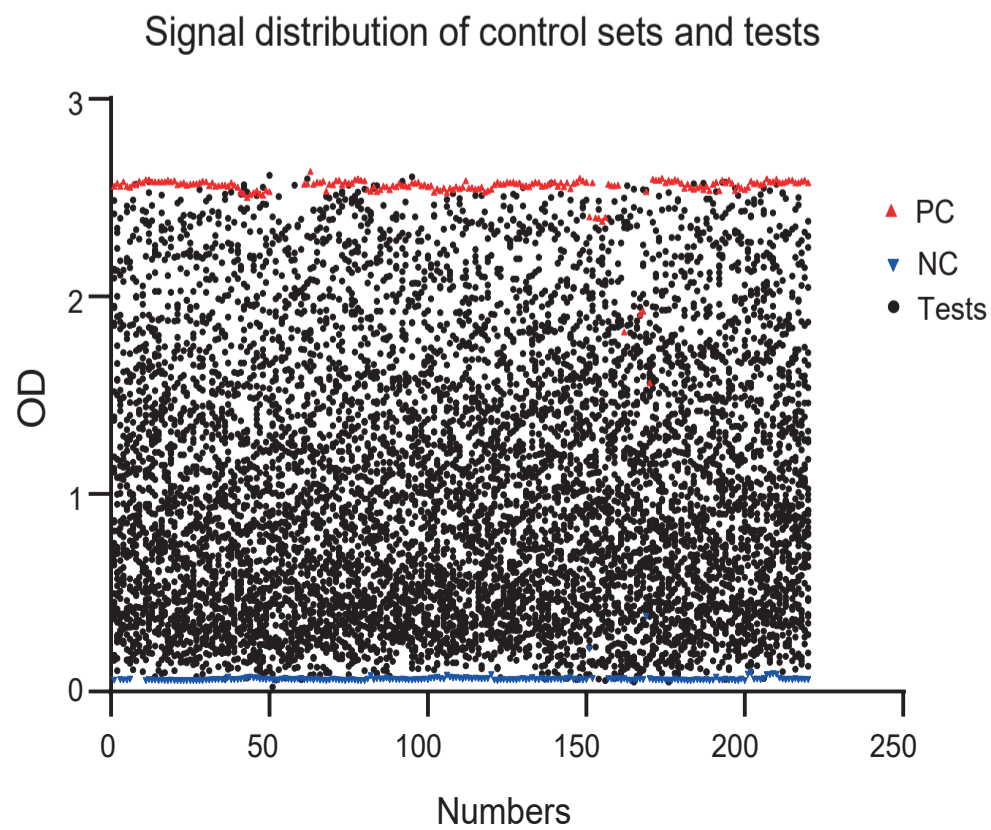

Supplement: qzae085_Supplementary_Data [file qzae085_supplementary_data.zip › FigureS5.pdf]

A

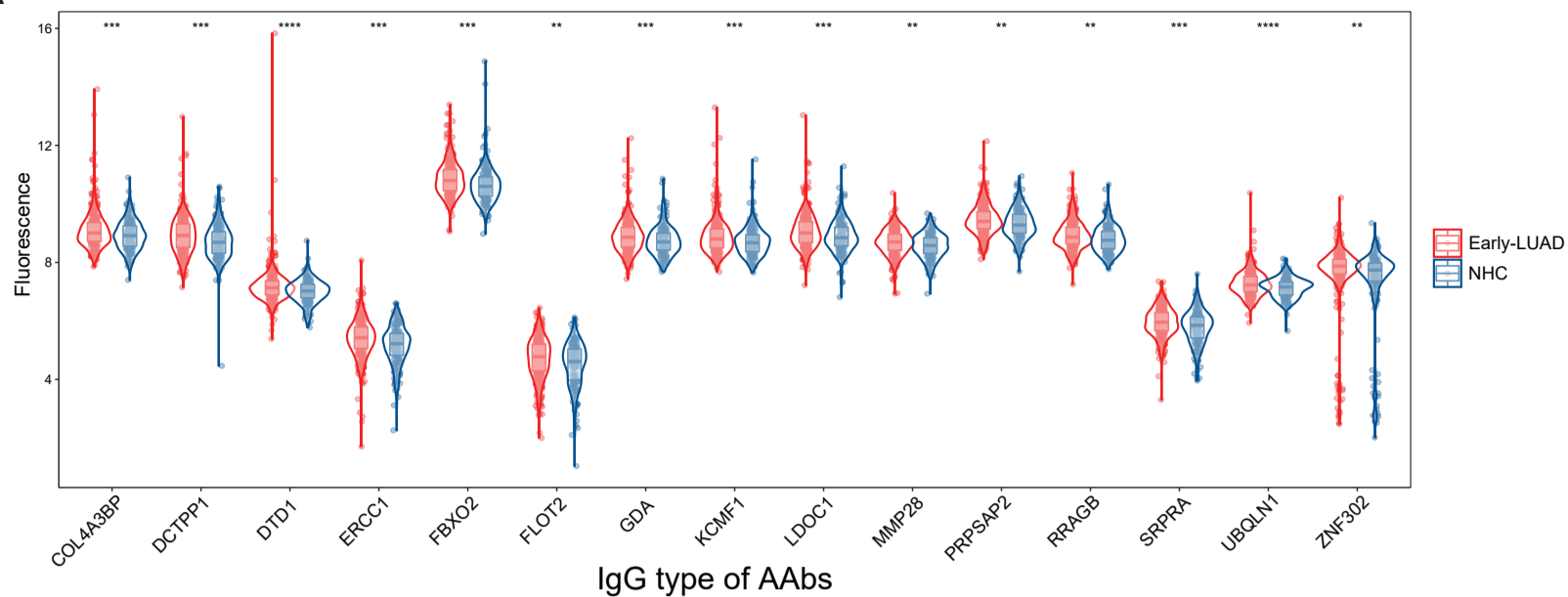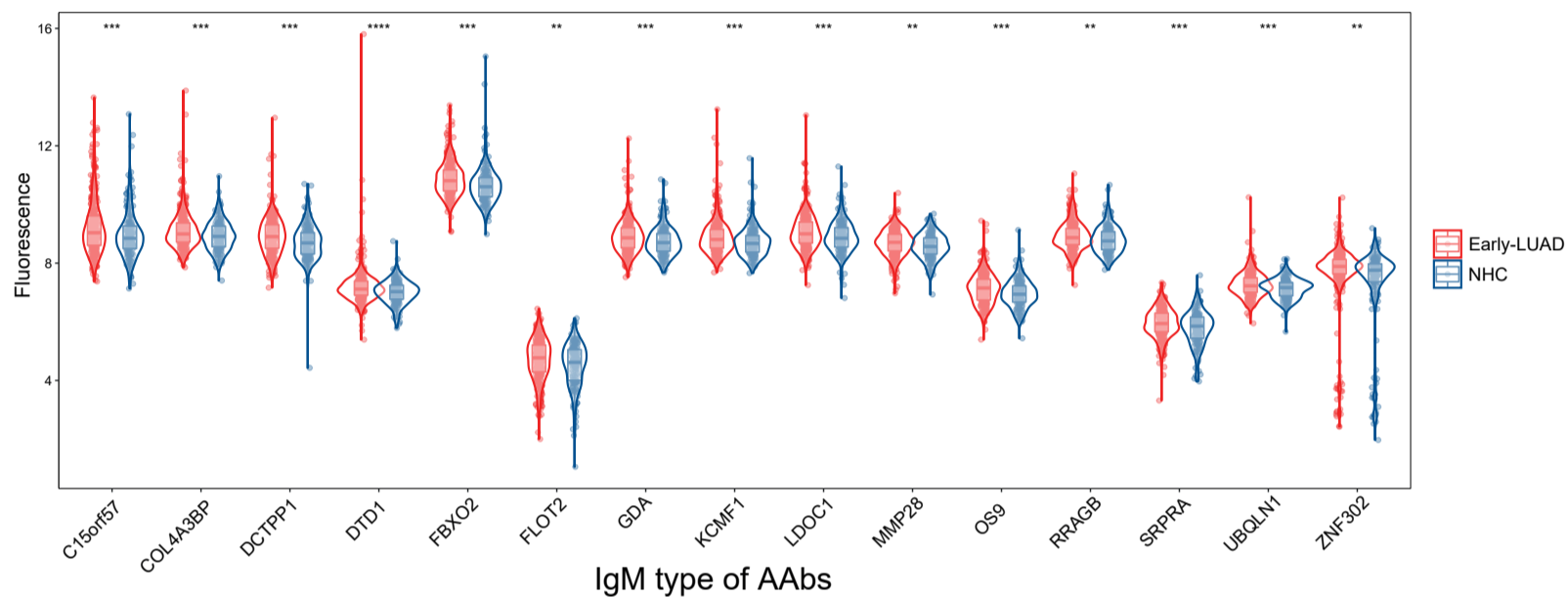

B

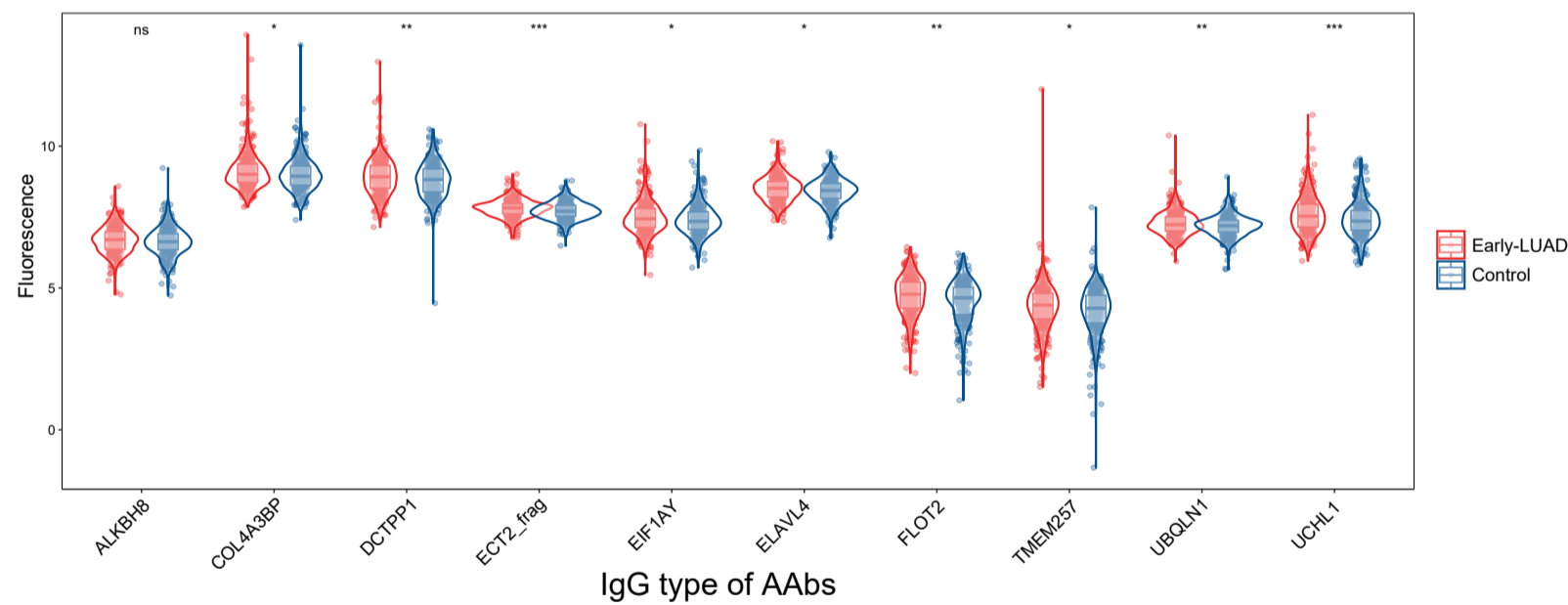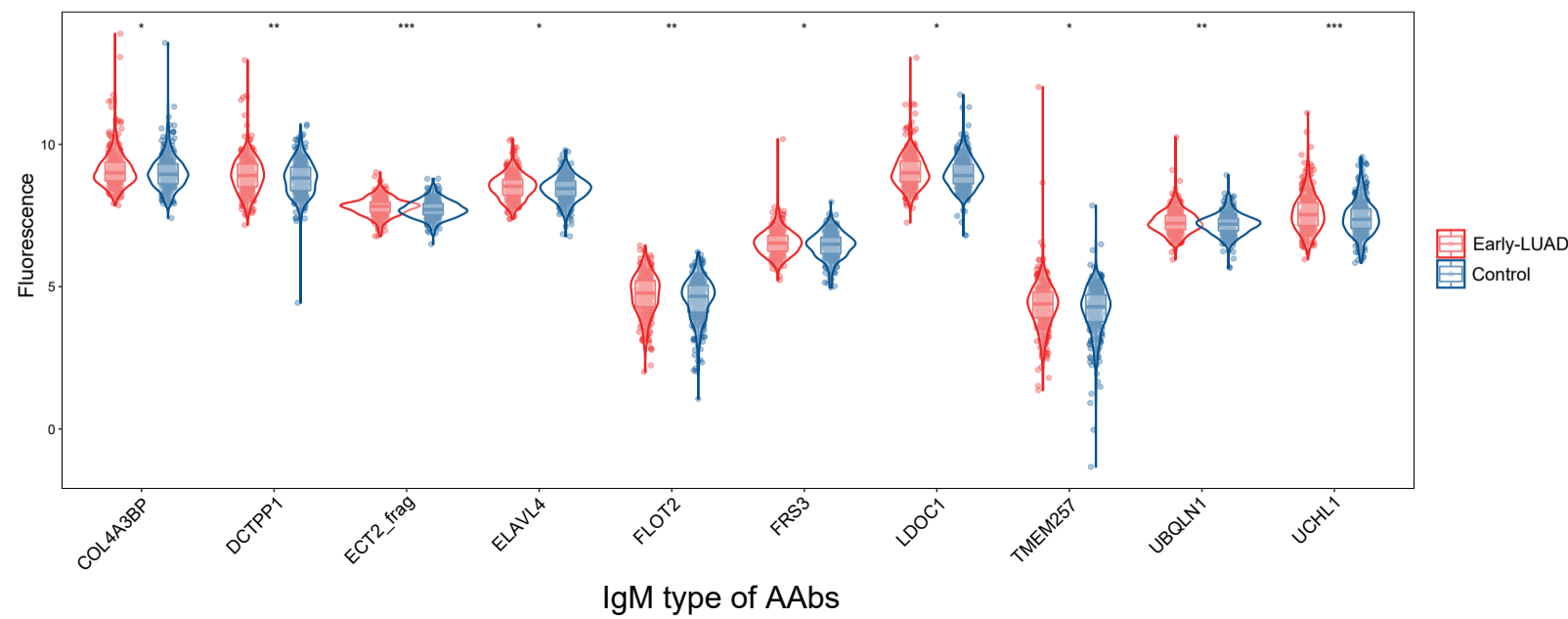

Supplement: qzae085_Supplementary_Data [file qzae085_supplementary_data.zip › Figure S4.pdf]

A

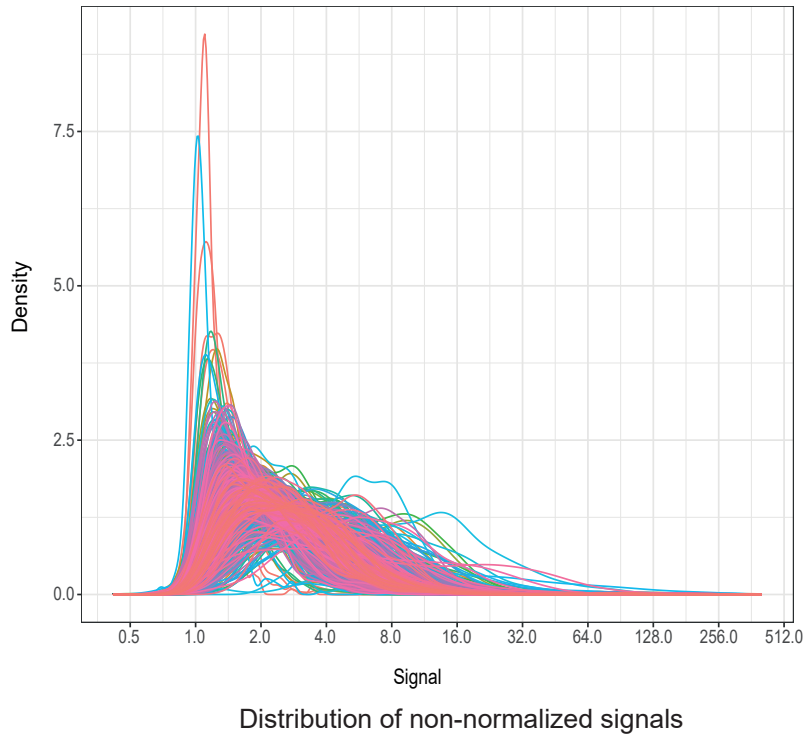

B

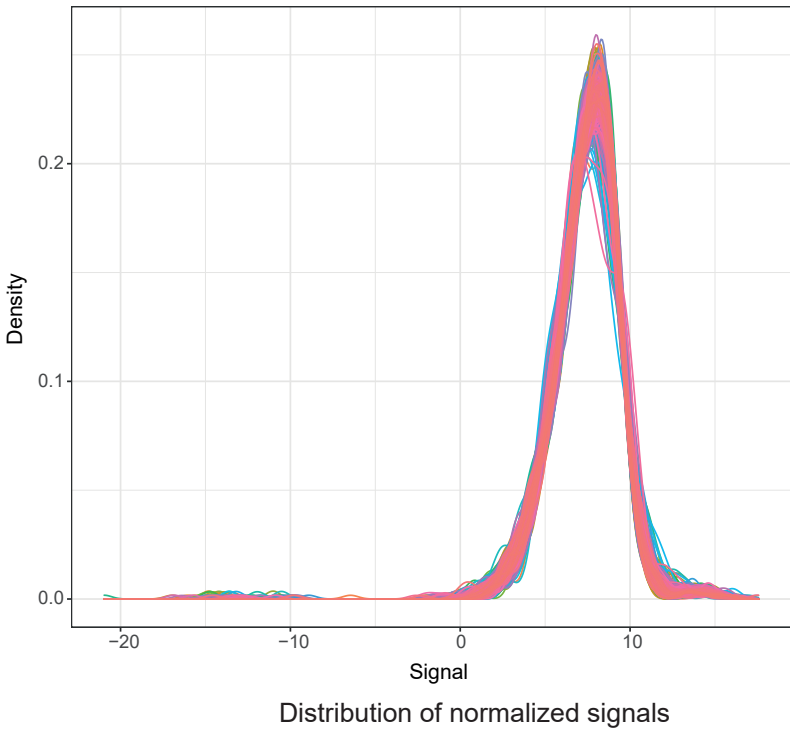

C

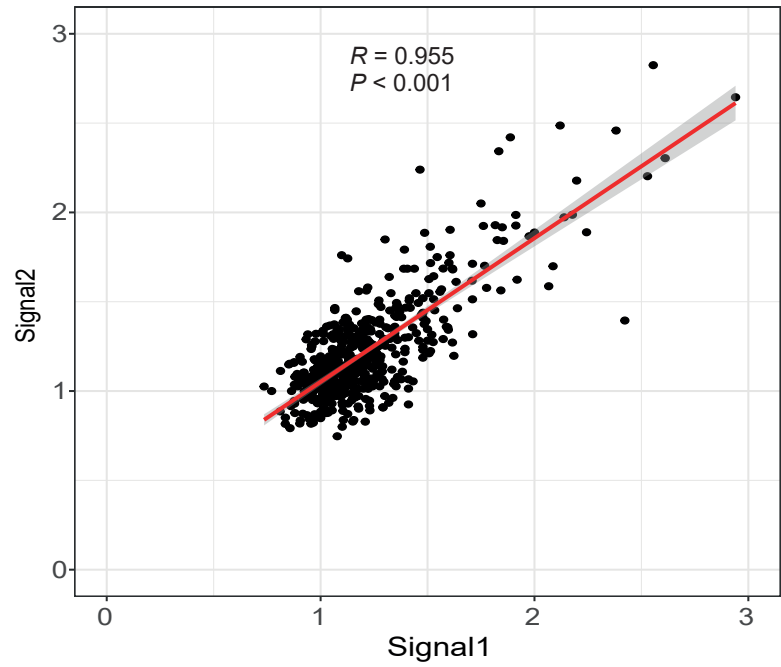

D

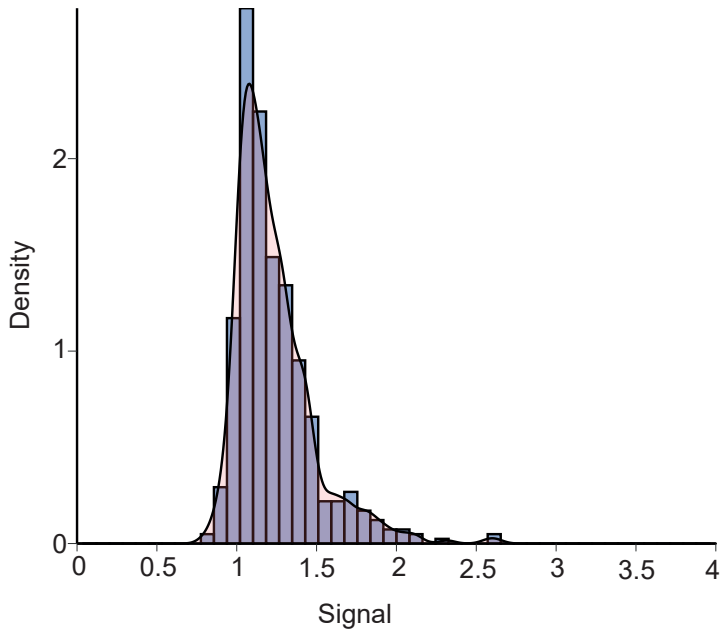

E

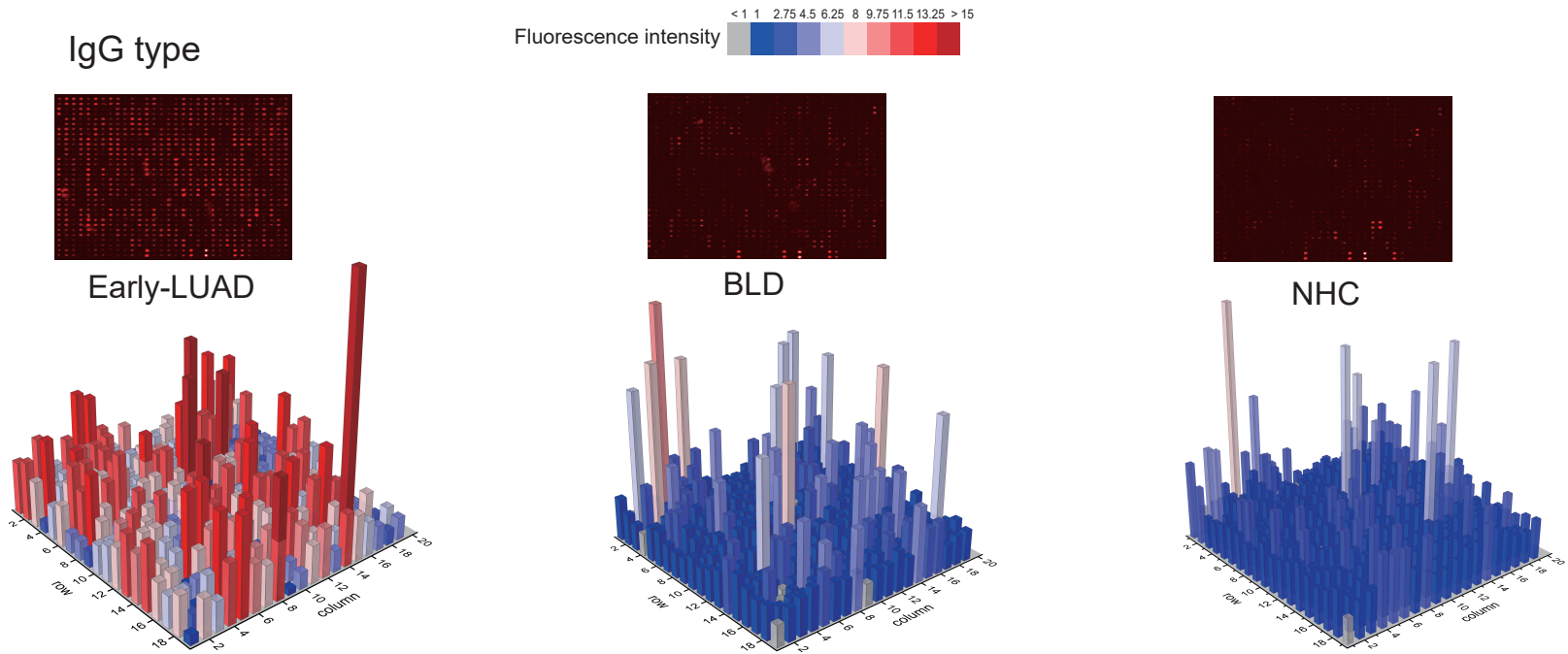

Supplement: qzae085_Supplementary_Data [file qzae085_supplementary_data.zip › FigureS3.pdf]
